# A Real‐World Analysis of Outcomes in CIC‐Rearranged Sarcomas: A Canadian Sarcoma Research and Clinical Collaboration (CanSaRCC) Study

**DOI:** 10.1002/cam4.71495

**Published:** 2026-01-19

**Authors:** Talya Wittmann Dayagi, Hagit Peretz Soroka, Alannah Smrke, Rebecca J. Deyell, Xiaolan Feng, Sapna Oberoi, Shantanu Banerji, Jonathan Noujaim, Nicolas Prud'homme, Ramy Saleh, Omar Farooq Khan, Jonathan Willard Bush, Bilal Marwa, Geoffrey Watson, Caroline Holloway, Lingxin Zhang, Abha Anshu Gupta, Jack Brzezinski

**Affiliations:** ^1^ Division of Haematology and Oncology, Department of Pediatrics, the Hospital for Sick Children University of Toronto Toronto Ontario Canada; ^2^ Division of Medical Oncology, Princess Margaret Cancer Centre University of Toronto Toronto Ontario Canada; ^3^ Department of Medical Oncology, BC Cancer University of British Columbia Vancouver British Columbia Canada; ^4^ Division of Pediatric Hematology/Oncology/BMT, B.C. Children's Hospital and Research Institute University of British Columbia Vancouver British Columbia Canada; ^5^ Division of Medical Oncology Tom Baker Cancer Center Calgary Alberta Canada; ^6^ Arthur Child Comprehensive Cancer Centre Calgary Alberta Canada; ^7^ Department of Pediatrics and Child Health, Max Rady College of Medicine University of Manitoba Winnipeg Manitoba Canada; ^8^ Department of Pediatric Hematology‐Oncology, CancerCare Manitoba University of Manitoba Winnipeg Manitoba Canada; ^9^ Department of Internal Medicine, Rady Faculty of Medicine, CancerCare Manitoba University of Manitoba Winnipeg Manitoba Canada; ^10^ Division of Medical Oncology, Hôpital Maisonneuve Rosemont University of Montreal Montreal Quebec Canada; ^11^ Division of Medical Oncology Centre Hospitalier Universitaire Sainte‐Justine Montreal Quebec Canada; ^12^ Department of Medical Oncology, McGill University Health Center McGill University Montreal Quebec Canada; ^13^ Division of Medical Oncology, Department of Oncology, Cumming School of Medicine University of Calgary Calgary Alberta Canada; ^14^ Department of Pathology and Laboratory Medicine, BC Children's Hospital University of British Columbia Vancouver British Columbia Canada; ^15^ Department of Pediatrics and Oncology University of Saskatchewan, Saskatchewan Cancer Agency, Saskatchewan Health Authority Saskatoon Saskatchewan Canada; ^16^ Department of Medical Oncology Mount Sinai Hospital Toronto Ontario Canada; ^17^ Division of Radiation Oncology, BC Cancer University of British Columbia Vancouver British Columbia Canada; ^18^ Department of Pathology and Laboratory Medicine, Mount Sinai Hospital University of Toronto Toronto Ontario Canada; ^19^ Cancer and Blood Disorders Center Seattle Children's Hospital Seattle Washington USA; ^20^ Department of Pediatrics University of Washington Seattle Washington USA

**Keywords:** adolescent and young adult oncology, chemotherapy response, CIC‐rearranged sarcoma, Ewing‐like sarcoma, metastatic sarcoma, soft‐tissue sarcoma, surgical resection

## Abstract

**Introduction:**

CIC‐rearranged sarcomas (CRS) are rare tumors predominantly affecting young adults. Despite distinct biology, CRS are often treated like Ewing sarcomas with multi‐agent chemotherapy, surgery, and radiotherapy, although the benefits of each remain unclear. We report the impact of treatment on response and survival.

**Methods:**

This retrospective multicenter study included patients diagnosed with CRS between 2002 and 2023. Demographic, treatment, and outcome data were collected. Event‐free (EFS) and overall survival (OS) were estimated using Kaplan–Meier analysis; risk factors were assessed via log‐rank tests and Cox regression. Treatment response was assessed using RECIST.

**Results:**

Among 27 patients (median age 21.5 years; range 8–83), 14 (52%) had localized and 12 (44%) metastatic disease (1 unknown). In the localized group, 6 (43%) received chemotherapy (Response: 2 complete, CR; 1 partial, PR; 1 mixed, MR; 1 stable, SD; 1 progressive disease, PD); 5 (36%) died—3 (21%) had received chemotherapy. Among metastatic patients, 10 (83%) received chemotherapy (1 CR, 5 PR, 2 MR, 1 PD); 9 died (one without chemotherapy). Local control was achieved in 12/14 (86%) localized and 7/12 (56%) metastatic patients. Only surgical resection was associated with improved OS (HR 0.13; *p* < 0.01) and EFS (HR 0.26; *p* < 0.01), particularly with R0 resection (EFS HR 0.23; *p* = 0.02). At median follow‐up of 18 months, 2‐year EFS was 56% vs. 8% (*p* = 0.02) and OS was 67% vs. 23% (*p* = 0.02) for localized vs. metastatic disease.

**Conclusion:**

Complete resection is critical in CRS. Chemotherapy benefit remains uncertain, underscoring the need for improved local control and novel, biology‐driven therapies.

## Graphical abstract

Chemotherapy was not associated with improved survival in localized or metastatic CIC‐rearranged sarcoma. Optimizing local control remains essential, and novel systemic therapies are urgently needed.

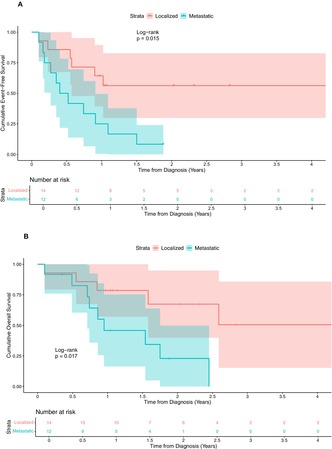

## 1 Introduction

Soft‐tissue sarcomas are a heterogeneous group of malignancies of mesenchymal origin, representing approximately 1% of all malignant tumors [1, 2]. *Capicua transcriptional repressor* (*CIC*)‐rearranged sarcomas (CRS), a subtype of small round blue cell sarcomas, were historically classified as undifferentiated small round cell or Ewing‐like sarcomas. However, the 2020 WHO classification recognized CRS as a distinct entity based on unique molecular and clinicopathologic characteristics [3, 4].

In the majority of cases (~95%), the *CIC* gene (19q13) is fused with one of two double‐homeobox (*DUX4*) retro‐genes (4q35 or 10q26), giving rise to the *CIC::DUX4* fusion. These tumors are rare—accounting for fewer than 1% of all sarcomas—and highly aggressive [5]. Additional, less common *CIC* fusion partners have been described, including *AXL*, *CITED1*, *LEUTX*, *SYK*, *FOXO4*, *NUTM1*, and *NUTM2A* [3, 6].

CRS primarily affects young adults (3rd to 4th decade of life), though the age spectrum spans from childhood to late adulthood [7, 8, 9, 10, 11]. Primary tumors often arise in the soft tissues of the head and neck, retroperitoneum, or pelvis, but may also involve viscera and/or bone. Metastatic disease at diagnosis is common, seen in over 40% of patients in published cohorts, with a predilection for the lungs [7, 8, 9, 12, 13].

Despite distinct biology from Ewing sarcoma, CRS is frequently managed with similar multi‐agent chemotherapy regimens. However, unlike for Ewing sarcoma [2, 14], the benefit of systemic therapy in CRS remains unclear, and early relapse or progression is common even among patients treated with multi‐agent chemotherapy [13, 15].

We conducted a retrospective study across Canadian academic centers to describe real‐world treatment patterns and outcomes for CRS. We aimed to evaluate outcomes in patients with localized and metastatic CRS, characterize systemic therapy use, and explore associations between treatment modality and survival. Our goal was to generate exploratory evidence to inform future efforts in this ultra‐rare sarcoma subtype.

## 2 Materials and Methods

### 2.1 Study Design, Participants, and Ethics Approval

This was a retrospective, multicenter cohort study conducted through the Canadian Sarcoma Research and Clinical Collaboration (CanSaRCC) database. Research ethics board (REB) approval was obtained at each of the 11 participating academic centers: The Hospital for Sick Children (Toronto), Princess Margaret Cancer Centre (Toronto), Mount Sinai Hospital (Toronto), BC Children's Hospital (Vancouver), BC Cancer (British Columbia), Hôpital Maisonneuve Rosemont (Montreal), Centre Hospitalier Universitaire Mère‐Enfant Sainte‐Justine (Montreal), the McGill University Health Centre (Montreal), Alberta Health Services (Edmonton and Calgary), CancerCare Manitoba (Winnipeg) and the Saskatchewan Health Authority (Saskatchewan). All active patients provided written informed consent and waiver of consent was REB‐approved for patients who were deceased or lost to follow up.

Patients diagnosed with CRS between 2002 and 2023 were eligible for inclusion. Diagnosis was made by expert sarcoma pathologists at participating centers based on histologic and immunophenotypic features, and where available, supported by molecular confirmation of *CIC*‐rearrangement. *CIC:DUX4* fusions were confirmed by Clinical Laboratory Improvement Amendments (CLIA)‐certified laboratories in the majority of cases; for others, the fusion partner was unknown, but clinical and histologic features were consistent with CRS.

### 2.2 Treatment Efficacy and Endpoints

Demographic, clinical, and pathological data were extracted from institutional medical records, including tumor size, anatomical location, fusion partner (if available), stage at diagnosis (localized or metastatic), treatment details (chemotherapy cycles, agents and doses; surgery type and margin status; radiotherapy dose, field, and timing), and clinical outcomes. Surgical margins were categorized as R0 (negative) or R1 (microscopically positive) per pathology reports [16]. Information on tumor depth (superficial vs. deep) was not uniformly available.

The primary outcomes were event‐free (EFS) and overall survival (OS). EFS was defined from date of diagnosis to first event—relapse, progression, or death, whichever occurred first. OS was defined from diagnosis to death from any cause, with censoring at last known follow‐up. Radiologic response was a secondary outcome, assessed locally at each participating center by sarcoma oncologists using RECIST v1.1 in a retrospective fashion and classified as complete response (CR), partial response (PR), stable disease (SD), or progressive disease (PD) [17]. Mixed response (MR) was defined as discordant changes among lesions, adjudicated within the framework of existing RECIST v1.1 rules.

### 2.3 Statistical Analysis

Descriptive statistics were utilized to summarize clinical and treatment characteristics. Between‐group comparisons were conducted using Fisher's exact test and Mann–Whitney *U* test for categorical and continuous variables, respectively.

Time‐to‐event outcomes (EFS and OS) were estimated using Kaplan–Meier analysis, and differences between subgroups were explored using the log‐rank test. Univariable Cox proportional hazards regression was performed to identify associations between clinical variables and survival outcomes. A limited multivariable model adjusting for metastatic status was also performed where appropriate, though statistical power was limited due to sample size. All analyses were considered exploratory and hypothesis‐generating. A *p*‐value < 0.05 was considered statistically significant. Analyses were performed using SPSS version 25 and R version 4.3.3.

## 3 Results

### 3.1 Patient Characteristics

Table 1 summarizes baseline characteristics and treatment, stratified by metastatic status. Among 27 included patients, median age at diagnosis was 21.5 years (range 9–83; interquartile range 15–35); the male‐to‐female ratio was 1.25:1. *CIC:DUX4* fusion was confirmed in 20 patients (74%), while the fusion partner was unknown in 7 (26%). Fourteen (52%) presented with localized and 12 (44%) with metastatic disease (1 unknown). Among metastatic cases, 8 (67%) had lung‐only involvement, while 4 (33%) had additional sites, including regional lymph nodes (*n* = 3), tumor thrombus (*n* = 1), bone (*n* = 1), and abdominal cavity (*n* = 1).

**TABLE 1:** Demographic, clinical, and treatment characteristics of patients with CIC‐rearranged sarcoma, stratified by metastatic status at diagnosis.

	Metastatic disease at diagnosis (%)	Localized disease at diagnosis (%)	Total (%)	*p*
Total	12 (44)	14 (52)	27^a^ (100)	—
Male	9 (75)	5 (36)	15 (56)	0.06
Median (range) age at diagnosis (years)	21.2 (12–40)	22.5 (8–83)	21.5 (8–83)	0.68
Location of primary tumor				0.02
Central	3 (25)	10 (71)	13 (48)	
Extremity	9 (75)	4 (29)	14 (52)	
Median maximal diameter of primary tumor	10.2 (2.5–30)	5.8 (2–30)	7.3 (2–30)	0.04
> 5 cm	8 (67)	7 (50)	15 (56)	0.18
≤ 5 cm	1 (8)	5 (36)	6 (22)	
NA	3 (25)	2 (14)	6 (22)	
*CIC* fusion partner				0.67
*DUX4*	8 (67)	11 (79)	20 (74)	
Other/unknown	4 (33)	3 (21)	7 (26)	
Metastatic disease at diagnosis			12 (44); 1 NA	—
Lung‐only	8 (67)	—	8 (67)	
Other ± Lung	4 (33)	—	4 (33)	
Chemotherapy given – Yes	10 (83)	6 (43)	16 (59)	0.05
1 line only	4 (33)	4 (29)	8 (30)	
2 or more lines	6 (50)	2 (14)	8 (30)	
First‐line chemotherapy regimen^b^				0.89
VDC/IE	5 (50)	3 (43)	8 (50)	
VAC	—	—	—	
IE	1 (10)	—	1 (6)	
VDC	1 (10)	—	1 (6)	
ID	2 (20)	1 (14)	3 (19)	
VACD	—	1 (14)	1 (6)	
ViT	1 (10)	1 (14)	2 (13)	
Best response to first‐line chemotherapy^b^				0.78
SD	—	1 (17)	1 (6)	
CR	1 (10)	2 (33)	3 (19)	
PR	5 (50)	1 (17)	6 (38)	
MR	2 (20)	1 (17)	3 (19)	
PD	1 (10)	1 (17)	2 (13)	
Unknown	1 (10)	—	1 (6)	
Surgery and/or RT (for primary tumor)	7 (58)	12 (86)	19 (70); 1 NA	0.55
RT alone	3 (25)	3 (21)	6 (22)	
Surgery alone	2 (17)	5 (36)	7 (26)	
Both RT and Surgery	2 (17)	4 (29)	6 (22)	
Local control – Surgical margins				0.05^c^
R0	2 (50)	8 (89)	10 (77)	
R1	2 (50)	1 (11)	3 (23)	

Abbreviations: A, Actinomycin D; C, Cyclophosphamide; cm, centimeters; CR, complete response; D, Doxorubicin; E, Etoposide; I, Ifosfamide; i, irinotecan; MR, mixed response; NA, not available; PD, progressive disease; PR, partial response; R0, negative surgical margins; R1, positive surgical margins; RT, radiotherapy; SD, stable disease; T, Temozolomide; V, Vincristine.

^a^ One patient had unknown metastatic status at diagnosis and is excluded from stratified analyses where applicable.

^b^ Percentages for chemotherapy regimens and responses are calculated based on patients who received systemic therapy.

^c^ *p*‐value compares R0 vs. R1/no resection.

**FIGURE 1:**
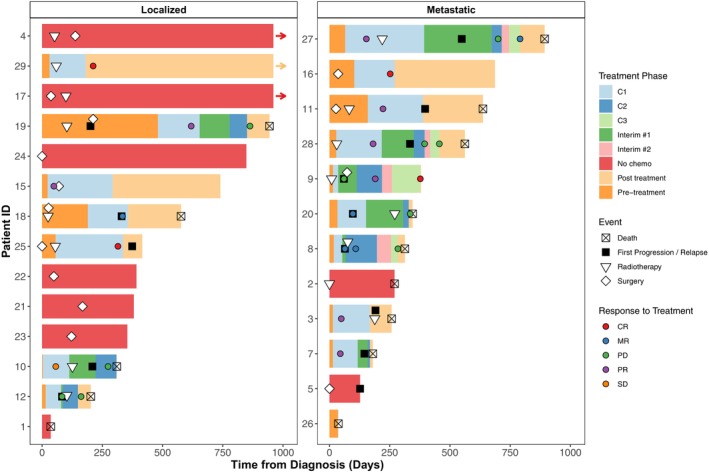
Longitudinal treatment and response overview in patients with CIC‐rearranged sarcoma. Swimmer's plot illustrating treatment timelines, response and survival status, stratified by metastatic status at diagnosis. *Arrows* denote patients with follow‐up exceeding 1000 days. *Pre‐treatment* refers to the interval between diagnosis and initiation of first‐line chemotherapy. *Interim #1* and *Interim #2* indicate the periods between first‐ and second‐line, and second‐ and third‐line chemotherapy, respectively, during which no systemic therapy was administered. *Post‐treatment* denotes the period following completion of all systemic therapy through last follow‐up. C1/C2/C3, first‐, second‐, and third‐line chemotherapy; CR, complete response; MR, mixed response; PD, progressive disease; PR, partial response; SD, stable disease.

A swimmer's plot (Figure 1) illustrates treatment courses and outcomes by metastatic status at diagnosis.

### 3.2 Management of Localized Disease at Diagnosis

Of the 14 patients with localized disease at diagnosis, 6 (43%) received chemotherapy at the time of diagnosis and one additional patient (Patient 19) received chemotherapy only after their first progression (Table 1). Among these, 3 received neo‐adjuvant chemotherapy: 1 received chemotherapy followed by surgery (R0 resection), 2 received chemotherapy followed by radiotherapy (RT). Of the remaining 3, 1 received chemotherapy alone with no surgery or RT, 3 received chemotherapy after surgery (R1 resection for both) and RT. First‐line regimens included alternating Vincristine (V)/Doxorubicin (D)/Cyclophosphamide (C) and Ifosfamide (I)/Etoposide (E) (*n* = 3; CR, PR and PD) (Patients 25, 15, 12), VC/Actinomycin (A) (*n* = 1, PR) (Patient 19), ID (*n* = 1, CR) (Patient 29), VACD (*n* = 1, SD) (Patient 10) and V/Irinotecan (i)/Temozolomide (T) (*n* = 1, MR) (Patient 18). Three patients received second‐line treatment including IE (*n* = 1, PD) (Patient 19), single agent V (*n* = 1, PD) (Patient 12), and oral topotecan (t) and pazopanib (*n* = 1, PD) (Patient 10).

Of the 8 patients who did not receive chemotherapy, local control included only surgery (all R0 resection) in 4, only RT in one, and both surgery and RT in 2. One patient died within 1 month of diagnosis, and it is unknown whether any treatment was administered.

### 3.3 Management of Metastatic Disease at Diagnosis

Among 12 patients with metastatic disease, 10 (83%) received chemotherapy (Table 1). First‐line regimens included alternating VDC/IE (*n* = 5; 1 CR, 2 PR, 2 MR) (Patients 16, 27, 28, 8, 20); IE alone (*n* = 1, PD) (Patient 9); VDC alone (*n* = 1, PR) (Patient 7); ID (*n* = 2; PR, unknown response) (Patients 3, 26); and V/iT (*n* = 1, PR) (Patient 11). One patient died of disease within 2 days of treatment initiation. Six patients received second‐line therapy—oral C (*n* = 1, unknown response) (Patient 7), ID (*n* = 1, PR) (Patient 9), V/iT (*n* = 1, MR) (Patient 8), VAC/IE (*n* = 1, PD) (Patient 28), and Ct (*n* = 2, PD) (Patients 20, 27)—and 4 received third‐line regimens: Vinorelbine and oral C (*n* = 1, PD) (Patient 8), Gemcitabine and Docetaxel (*n* = 1, MR) (Patient 27), iT (*n* = 1, PD) (Patient 28), and pegylated liposomal D (*n* = 1, given as maintenance, CR) (Patient 9).

Seven patients (58%) received local control prior to first progression or relapse: RT alone (*n* = 3), surgery alone (*n* = 2), or both (*n* = 2). Resection margins were R0 and R1 in 2 patients each. Three (25%) patients received RT for metastatic control.

### 3.4 Outcomes

In our full cohort (*N* = 27), median follow‐up for survivors was 18 months (range, 4–98). Fifteen patients (56%) have died, 14 of disease and 1 of unknown cause prior to treatment. Median time to death was 10 months (range 1–31 months; Table 2, Figure S1).

**TABLE 2:** Treatment outcomes, stratified by metastatic status at diagnosis.

	Metastatic disease at diagnosis (%) *N* = 12	Localized disease at diagnosis (%) *N* = 14	Total (%) *N* = 27^a^
Disease progression/relapse	9 (75); 1 NA	5 (36); 1 NA	14 (51); 3 NA
Local	1 (8)	1 (7)	2 (7)
Distant	5 (42)	4 (29)	9 (33)
Both	3 (25)	—	3 (11)
Status at last follow up			
Alive	3 (25)	9 (64)	12 (44)
NED	2 (17)	8 (57)	10 (37)
AWD	1 (8)	1 (7)	2 (7)
Median (range) follow up time in months	12 (4–23)	24 (12–98)	18 (4–98)
Deceased	9 (75)	5 (36)	15 (56)
Median (range) time from diagnosis to death in months	10 (1–29)	10 (1–31)	10 (1–31)

Abbreviations: AWD, alive with disease; NA, not available; NED, no evidence of disease.

^a^ One patient had unknown metastatic status at diagnosis and is excluded from stratified analyses where applicable.

#### 3.4.1 Outcomes of Localized Disease at Diagnosis

Among 14 patients with localized disease at diagnosis, median follow‐up for survivors was 24 months (range, 12–98; Table 2). Eight (57%) are alive without disease—7 of whom had R0 resection (1 neo‐adjuvant VDC/IE with PR, 2 also had RT—1 of whom with adjuvant VDC/IE); 1 with head/neck non‐*DUX4* CRS achieved CR with chemotherapy plus RT without surgery. One patient is alive with recurrent metastatic disease (14 months from diagnosis; 1.4 months from latest recurrence), and 5 (36%) have died.

Two patients received RT for local control, without surgical resection, along with chemotherapy—one of whom remained disease‐free over 5 years post treatment, while the other experienced disease progression and died. One patient received RT alone for local control, and only after relapse underwent surgery (R1) and received chemotherapy but continued to progress and subsequently died.

Two‐year EFS and OS were 56% (95% CI, 30%–83%) and 67% (95% CI, 40%–95%), respectively. No deaths occurred among localized patients with R0 resection (0/8), compared to 5/6 patients (83%) with incomplete resection (R1) or no surgery (*p* < 0.01). Death occurred in 3/6 (50%) chemotherapy recipients and 2/8 non‐recipients (25%, *p* = 0.58). Local recurrence (LR) occurred in 1/5 (20%) with non‐R0 resection and in none of the R0 cases.

#### 3.4.2 Outcomes of Metastatic Disease at Diagnosis

Among 12 patients with metastatic disease at diagnosis, median follow‐up for survivors was 12 months (range, 4–23; Table 2); median OS was 11 months. One patient (8%) remains alive with recurrent metastatic disease (lost to follow up 4 months from diagnosis; progressed post primary mass R0 resection; no chemotherapy), and 9 (75%) have died. Two patients (17%) remain alive with no evidence of disease at data cut‐off at 23 and 11 months from diagnosis. Both presented with lung‐only metastases: an adult with non‐*DUX4* CRS, who underwent surgery (R1) and had CR to adjuvant chemotherapy (VDC/IE), currently 23 and 14 months from diagnosis and end of treatment, respectively; and an adult with *DUX4*‐positive CRS, who had a PD on first‐line IE regimen and RT, R0 resection of primary mass, lung metastatic relapse followed by a PR on second‐line ID regimen, and finally a CR to radiosurgery, currently on maintenance pegylated liposomal Doxorubicin, 13 and 11 months from diagnosis and last recurrence, respectively.

Two‐year EFS was 8% (95% CI, 0%–24%) and OS was 23% (95% CI, 0%–50%). Three patients received RT for local control, without surgical resection—all three ultimately died despite chemotherapy. Eight of the 10 patients who received chemotherapy (80%) died while 1/2 non‐recipients (50%, *p* = 0.45) died and the other was lost to follow up at 4 months. LR occurred in 2/8 (25%) after R1 resection and in 1/2 (50%) after R0 resection.

#### 3.4.3 Survival Analysis

**FIGURE 2:**
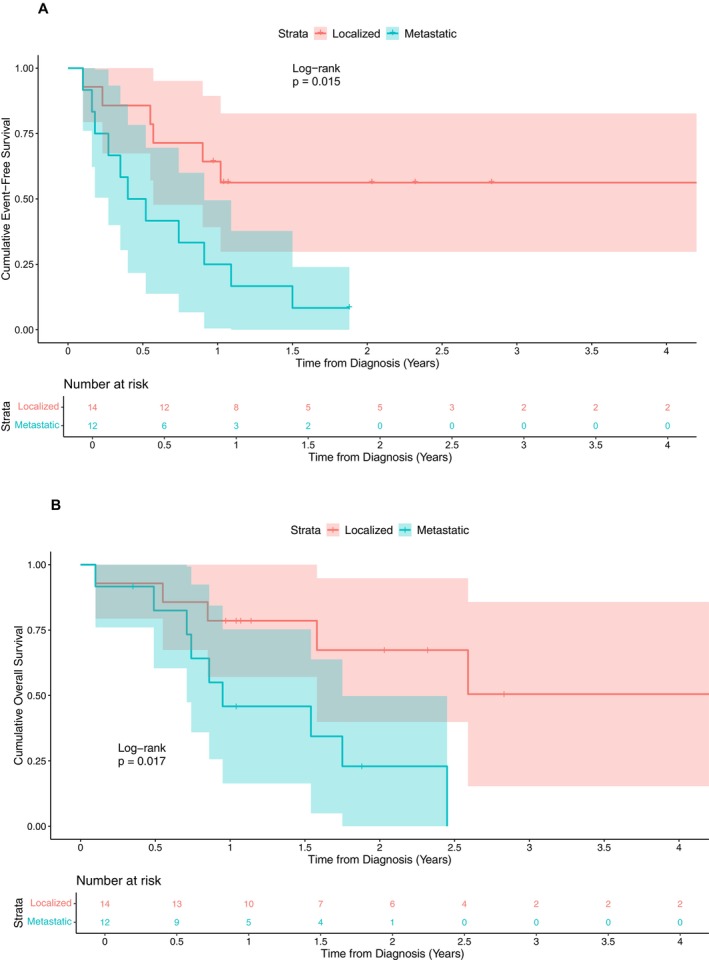
Kaplan–Meier survival analysis stratified by metastatic status at diagnosis. (A) EFS: 2‐year EFS was 56% (95% CI 30%–83%) for patients with localized disease and 8% (95% CI 0%–24%) for those with metastatic disease (*p* = 0.02). (B) OS: 2‐year OS was 67% (95% CI 40%–95%) for patients with localized disease and 23% (95% CI 0%–50%) for those with metastatic disease (*p* = 0.02). Tick marks indicate censored observations. CI, confidence interval; EFS, Event‐free survival; OS, Overall survival.

Two‐year EFS and OS were worse in patients with metastatic vs. localized disease at diagnosis (log‐rank test, *p* = 0.02 and *p* = 0.02, respectively; Table S1, Figure 2).

There was no significant association between *CIC* fusion partner and metastatic status at diagnosis (*p* = 0.67) or survival (*p* = 0.66).

Two‐year EFS and OS were significantly better in patients who underwent complete surgical resection, compared to those who did not, for the whole cohort; EFS was 57% (95% CI 27%–87%) vs. 8% (95% CI 0%–22%, *p* < 0.01) and OS was 71% (95% CI 38%–100%) vs. 23% (95% CI 0%–46%, *p* < 0.01), respectively (Table S1, Figures S2B, S3B).

In exploratory Cox regression (Table 3), metastatic disease at diagnosis was associated with increased risk of event (HR 3.24, 95% CI 1.19–8.83; *p* = 0.03) and death (HR 3.82; 95% CI 1.16–12.58; *p* = 0.03). Local control of any modality (surgical resection and/or RT) was associated with lower risk of event (HR 0.15, 95% CI 0.05–0.44; *p* < 0.01) and death (HR 0.06, 95% CI 0.02–0.26; *p* < 0.01). Surgical resection was associated with improved EFS (vs no resection: HR 0.22, 95% CI 0.07–0.63; *p* < 0.01) and OS (vs no resection: HR 0.13, 95% CI 0.03–0.46; *p* < 0.01), particularly with R0 margins (EFS vs non‐R0 resection: HR 0.23, 95% CI 0.06–0.79; *p* = 0.02). Moreover, a definitive surgical resection remained significant even when adjusted for metastatic disease at diagnosis (EFS: 0.29, CI 0.09–0.92, *p* = 0.04; OS: HR 0.15, CI 0.03–0.68, *p* = 0.01). RT alone was not associated with a significant survival benefit (*p* = 1.0).

**TABLE 3:** Univariable Cox regression analysis of event‐free survival and overall survival in patients with CIC‐rearranged sarcoma.

Characteristics	EFS		OS	
	HR (95% CI)	*p*	HR (95% CI)	*p*
Metastatic disease at diagnosis	**3.24 (1.19–8.83)**	**0.03**	**3.82 (1.16–12.58)**	**0.03**
Chemotherapy given	2.00 (0.71–5.61)	0.19	2.13 (0.66–6.88)	0.21
	1.47 (0.42–5.16)*	0.55*	1.39 (0.29–6.68)*	0.68*
Local control with RT and/or surgery	**0.15 (0.05–0.44)**	**< 0.01**	**0.06 (0.02–0.26)**	**< 0.01**
	0.22 (0.06–0.79)*	0.02*	0.08 (0.02–0.41)*	< 0.01*
Local control with RT	1.02 (0.39–2.66)	0.97	0.52 (0.16–1.66)	0.27
	1.14 (0.44–2.99)*	0.79*	0.53 (0.16–1.70)*	0.28*
Local control with surgery	**0.22 (0.07–0.63)**	**< 0.01**	**0.13 (0.03–0.46)**	**< 0.01**
	**0.29 (0.09–0.92)**	0.04*	0.15 (0.03–0.68)*	0.01*
Clean(R0) surgical margins vs. R1 or no resection	**0.23 (0.06–0.79)**	**0.02**	0.02 (0.00–1.50)	0.08
	0.32 (0.07–1.51)*	0.15*	0.01 (0.00–4.55)*	0.15*
Relapse or progression	—	—	**13.65 (1.73–107.70)**	**< 0.01**
			**8.80 (1.01–76.41)**	0.05*

*Note:* HR with 95% CI and corresponding *p*‐values are presented for each variable. Where indicated (*), stratified models account for metastatic status at diagnosis, due to its prognostic impact. Clean surgical margins (R0) are compared with positive margins (R1) or no resection. Bolded values are statistically significant (*p* ≤ 0.05).

Abbreviations: CI, confidence interval; EFS, event‐free survival; HR, hazard ratio; OS, overall survival; R0, negative surgical margins; R1, microscopically positive surgical margins; RT, radiotherapy; vs., versus.

Chemotherapy did not appear to significantly impact EFS or OS (*p* = 0.19 and *p* = 0.21, respectively; Figures S2C, S3C, S5).

Consistent with the extent of disease at presentation, patients with metastatic disease were more likely to have non‐resectable tumors (defined as R1 resection or no surgery; *p* = 0.05). Patients who did not receive any form of local control had significantly lower 2‐year EFS and OS compared to those who did, even after adjusting for metastatic status at diagnosis (log‐rank test, *p* < 0.01 and *p* = 0.01, respectively). Definitive surgical resection was particularly associated with improved 2‐year EFS (*p* = 0.03) and OS (*p* < 0.01) after adjustment (Table S1, Figure S4).

## 4 Discussion

This retrospective multicenter study presents a national real‐world cohort of CRS patients with detailed treatment and outcome data across pediatric and adult institutions. Our primary aim was to describe outcomes in this ultra‐rare disease, with a particular focus on systemic therapy and surgical resection.

Despite distinct molecular features and generally poorer prognosis compared to ES, patients with CRS are frequently treated with ES‐based chemotherapy regimens, largely due to historical overlap in histology and presentation [18]. Our findings raise important questions about the applicability of this approach.

Outcomes for patients with metastatic disease at diagnosis remained poor irrespective of treatment; median OS was 11 months and 2‐year EFS and OS were only 8% and 23%, respectively in our series. Longer‐term survival was seen in 1 non‐DUX4 metastatic patient. Out of 16 patients who received chemotherapy, 11 had response to first line therapy (3/6 localized, 8/10 metastatic), but the majority of these patients eventually had a progression/relapse or death, with the exception of one patient with localized disease who had a sustained CR after radiation and chemotherapy (Patient 29) and one patient with metastatic disease who achieved CR after surgery and chemotherapy (Patient 16). Two patients had a “mixed response” wherein at least one lesion demonstrated PR and at least one demonstrated PD by RECIST criteria. This type of response is clinically relevant and different from either PR or PD in that it suggests the presence of different clones with different sensitivity within each lesion [19]. However, both of these patients eventually died of disease.

Our results align with prior retrospective series, where metastatic or locally advanced CRS at diagnosis has been almost uniformly lethal despite frequent chemotherapy use. In the French Sarcoma Group's series (all received chemotherapy), median OS was 10–15 months [20]. Connolly et al. found median OS was 12.6 months (86% received chemotherapy), with a single patient achieving a sustained complete response [9]. In the GRACefUl study, 3‐year OS was 20% [13]. Most recently, Murphy et al. reported a median OS of 16 months [21]. Together, these data reinforce that metastatic CRS remains a highly lethal diagnosis, with survival gains largely confined to exceptional cases.

In contrast, localized CRS carries a substantially more favorable prognosis, particularly when complete surgical resection is achieved. In our cohort, 2‐year OS was 67%, with no deaths among patients who underwent R0 resection, including one who achieved it following neo‐adjuvant chemotherapy (PR)—suggesting a potential role for chemotherapy in facilitating complete resection. Outcomes were notably worse in localized patients who did not achieve complete resection, underscoring the central role of local control—particularly definitive surgery in CRS management. These results align with Antonescu et al., who reported a 5‐year OS of 49% [7], Connolly et al. who found a median OS of 40.6 months (73% received chemotherapy) [9], and the GRACefUl study, where 3‐year OS was 56% [13]. Collectively, these findings demonstrate that long‐term survival is achievable in localized CRS—especially with R0 resection—highlighting the stark prognostic divide between localized and metastatic disease.

We performed exploratory Cox regression modeling to assess the impact of chemotherapy and local control on survival outcomes. Although sample size precluded full multivariable modeling with multiple covariates, surgical resection remained significantly associated with improved EFS and OS even after adjustment for metastatic status. These findings suggest a potential survival benefit with local control—particularly complete surgical resection—independent of metastatic disease. However, interpretation is limited by small cohort size and the exploratory nature of the analysis.

While RT alone has been shown to provide effective local control in ES and is often used for unresectable tumors [22, 23], it did not yield similar outcomes in CRS. In our cohort, no patients treated with RT alone were long‐term survivors, whereas complete surgical resection appeared essential for achieving favorable outcome. This aligns with our broader observation that CRS may require a fundamentally different treatment approach than ES.

Whether *CIC::DUX4* fusions confer different outcomes compared to other *CIC* fusions remains uncertain. Our data, like those of prior cohorts, were underpowered to detect significant prognostic differences between fusion partners.

Considering the rarity of CRS, our study was strengthened by using a national multicenter registry (CanSaRCC) to gather detailed data on patients with this ultra‐rare tumor, treated in real‐world clinical settings by specialist sarcoma teams [24, 25]. All diagnoses were rendered by sarcoma pathologists at major academic institutions. *CIC:DUX4* fusions were confirmed in 74% of patients; the remaining cases met histologic and clinical criteria consistent with CRS, despite missing fusion partner data.

Treatment decisions, including use and selection of chemotherapy, were made at the discretion of the treating institution and multidisciplinary team. Reasons for omitting systemic therapy were inconsistently documented but may have included advanced patient age, limited disease burden, comorbidities, or clinical judgment regarding limited expected benefit.

Limitations of this study include its retrospective design, small sample size, lack of centralized histologic and radiographic review, and missing data on response to local control. Tumor depth was not consistently documented, and treatment decisions were not standardized across institutions. Although both *DUX4* and non‐*DUX4* fusion partners were included to increase cohort size, the study was underpowered to reliably evaluate potential biological differences between them.

Given the uncertainty surrounding the benefit of cytotoxic chemotherapy, complete resection remains critical in CRS. The dismal outcomes for metastatic disease further underscore the need for improved local control and novel, biology‐driven therapies. Ongoing trials, as reviewed by Ponce et al., include agents such as Dinaciclib (CDK2 inhibitor), Adavosertib (WEE1 inhibitor), Linsitinib (IGF‐1 receptor inhibitor), iP300w (p300/CBP inhibitor), anti‐DUX4 antibodies, and BCI (DUSP6 inhibitor) for *CIC::DUX4* sarcomas [26]. In addition, emerging evidence highlights the role of epigenetic dysregulation in fusion‐driven sarcomas, including CRS, suggesting potential for therapies targeting chromatin remodeling and transcriptional regulation. Incorporating epigenomic profiling into future trials may help identify biomarker‐defined subsets more likely to respond to such strategies [27].

## 5 Conclusion

In conclusion, our findings highlight the critical role of complete surgical resection in achieving long‐term survival for patients with CRS, particularly in localized resectable disease. While our exploratory analysis did not demonstrate a clear survival benefit with cytotoxic chemotherapy in the management of local disease, we were not able to assess the relative impact of DUX4 positivity on outcomes. Optimizing local control should remain central to management, and future research should focus on multicenter efforts to biobank tissue, integrate molecular and epigenomic profiling, and accelerate the development of novel and targeted therapies for this rare and aggressive sarcoma.

## Author Contributions

**Talya Wittmann Dayagi:** conceptualization (equal), data curation (equal), formal analysis (equal), investigation (equal), visualization (lead), writing – original draft (lead), writing – review and editing (lead). **Hagit Peretz Soroka:** conceptualization (equal), data curation (lead), formal analysis (equal), investigation (equal), methodology (equal), project administration (equal), visualization (equal), writing – review and editing (equal). **Alannah Smrke:** data curation (equal), writing – review and editing (supporting). **Rebecca J. Deyell:** data curation (equal), writing – review and editing (supporting). **Xiaolan Feng:** data curation (equal), writing – review and editing (lead). **Sapna Oberoi:** data curation (equal), writing – review and editing (supporting). **Shantanu Banerji:** data curation (equal), writing – review and editing (supporting). **Jonathan Noujaim:** data curation (equal), writing – review and editing (supporting). **Nicolas Prud'homme:** data curation (equal), writing – review and editing (supporting). **Ramy Saleh:** data curation (equal), writing – review and editing (supporting). **Omar Farooq Khan:** data curation (equal), writing – review and editing (supporting). **Jonathan Willard Bush:** data curation (equal), writing – review and editing (supporting). **Bilal Marwa:** data curation (equal), writing – review and editing (supporting). **Geoffrey Watson:** data curation (equal), writing – review and editing (supporting). **Caroline Holloway:** data curation (equal), writing – review and editing (supporting). **Lingxin Zhang:** data curation (equal), writing – review and editing (supporting). **Abha Anshu Gupta:** conceptualization (equal), data curation (equal), funding acquisition (lead), investigation (equal), methodology (equal), supervision (equal), writing – review and editing (equal). **Jack Brzezinski:** conceptualization (equal), formal analysis (equal), investigation (equal), supervision (lead), visualization (equal), writing – review and editing (equal).

## Conflicts of Interest

The authors declare no conflicts of interest.

## Supporting information


**Figure S1:** Kaplan–Meier estimates of 2‐year event‐free and overall survival in patients with CIC‐rearranged sarcoma.

**Figure S2:** Kaplan–Meier analysis of 2‐year event‐free survival stratified by treatment modality.

**Figure S3:** Kaplan–Meier analysis of 2‐year overall survival stratified by treatment modality.

**Figure S4:** Kaplan–Meier estimates of 2‐year event‐free survival and overall survival stratified by definitive surgery, adjusted for metastatic status.

**Figure S5:** Kaplan–Meier estimates of 2‐year event‐free survival and overall survival stratified by chemotherapy use, adjusted for metastatic status.

**Table S1:** Kaplan–Meier estimates and log‐rank analysis of event‐free and overall survival in patients with CIC‐rearranged sarcoma.

## Data Availability

Access to specific data and analyses can be requested through cansarcc@uhn.ca.
