# Supplementary figures and images for: A Real‐World Analysis of Outcomes in CIC‐Rearranged Sarcomas: A Canadian Sarcoma Research and Clinical Collaboration (CanSaRCC) Study
Source: Cancer Med. 2026 Jan 19;15(1):e71495. doi: 10.1002/cam4.71495 (PMC12815609; doi:10.1002/cam4.71495)

**B**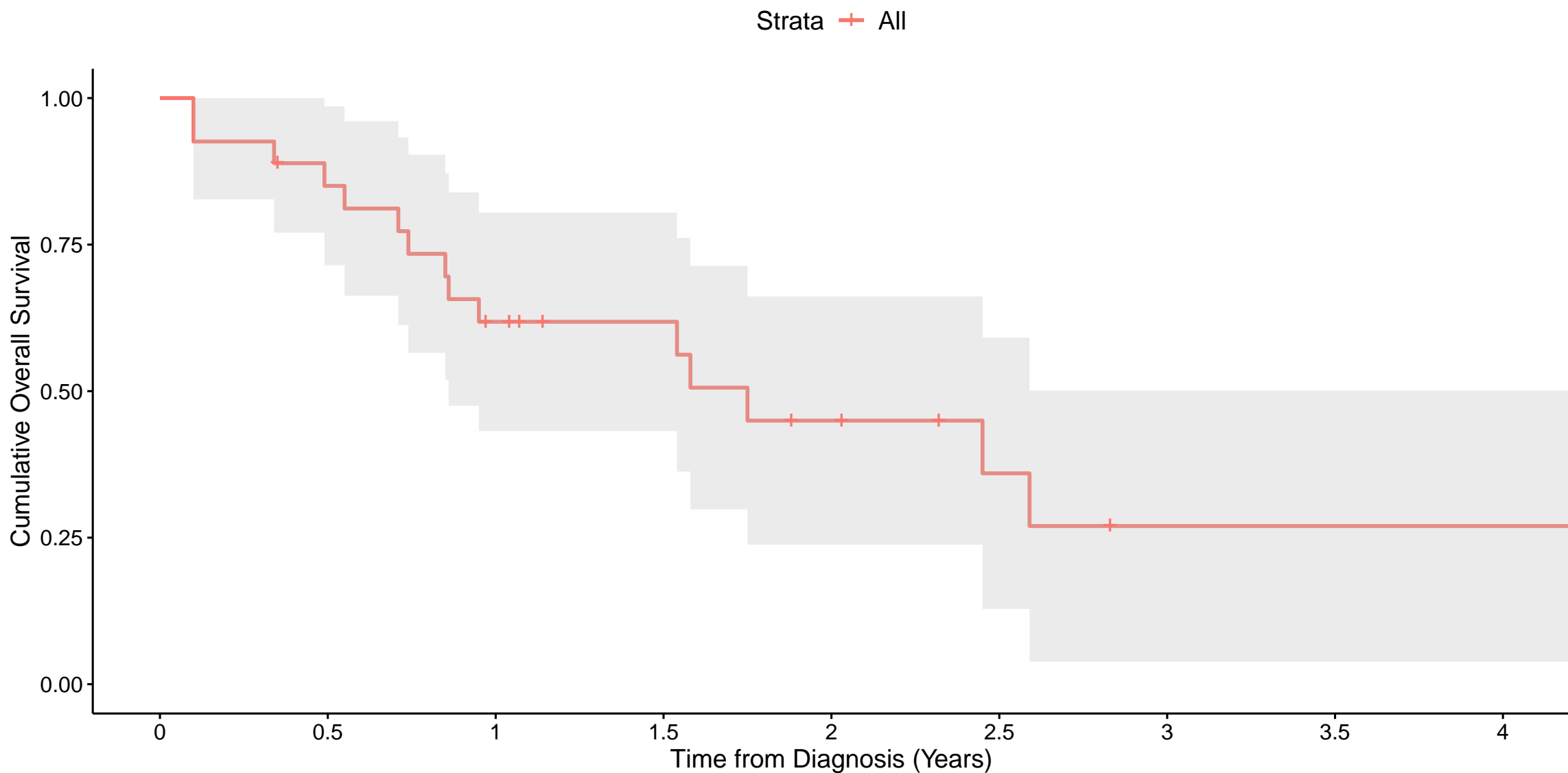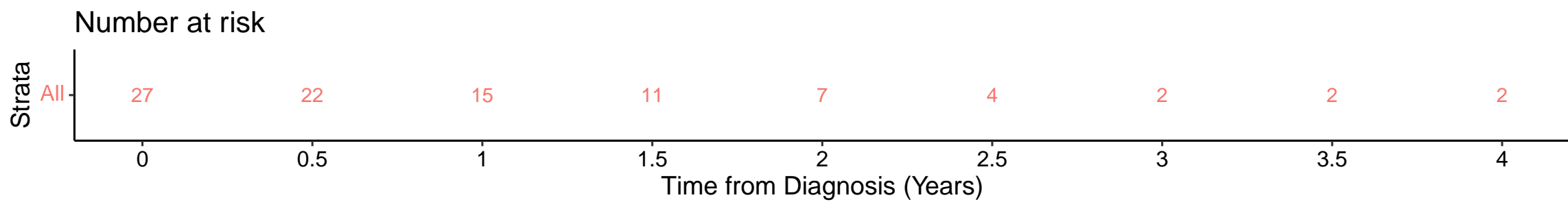

Supplement: Supplementary file 1 — Figure S1: Kaplan–Meier estimates of 2‐year event‐free and overall survival in patients with CIC‐rearranged sarcoma. [file CAM4-15-e71495-s006.zip › cam471495-sup-0002-FigureS1@suppfig1B.pdf]

**A**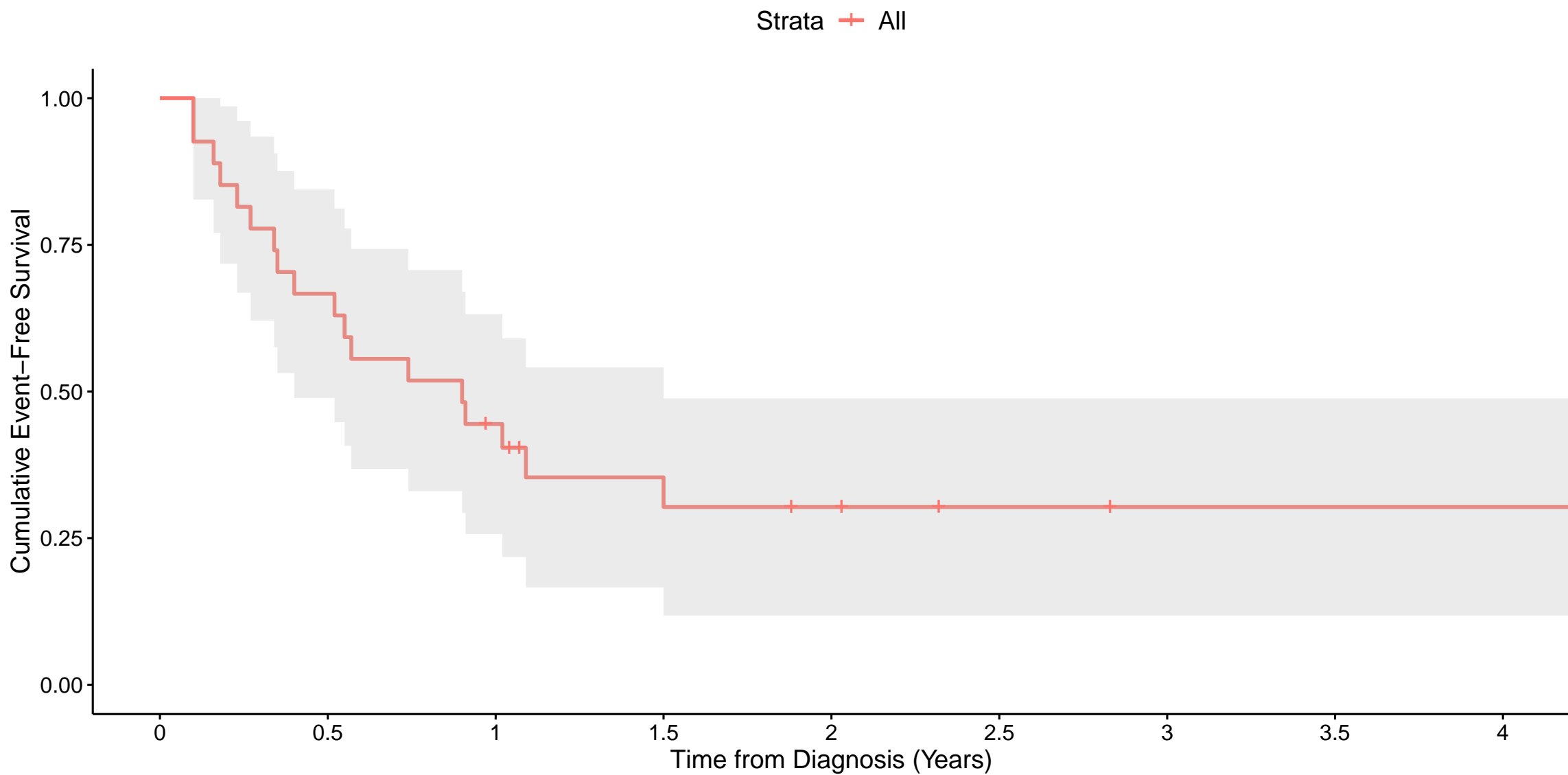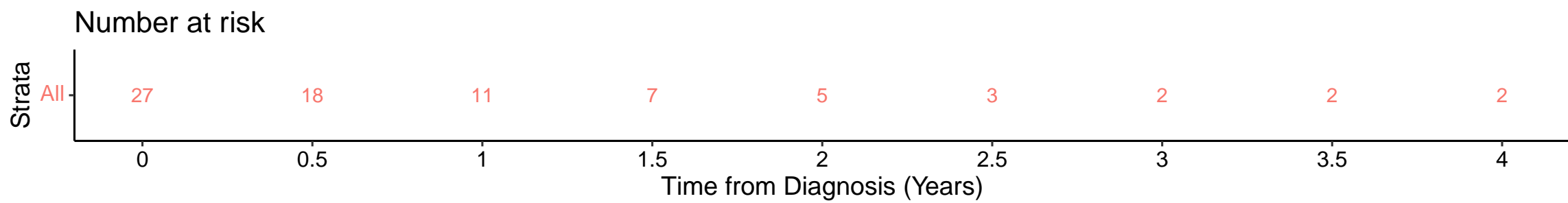

Supplement: Supplementary file 1 — Figure S1: Kaplan–Meier estimates of 2‐year event‐free and overall survival in patients with CIC‐rearranged sarcoma. [file CAM4-15-e71495-s006.zip › cam471495-sup-0001-FigureS1@suppfig1A.pdf]

A

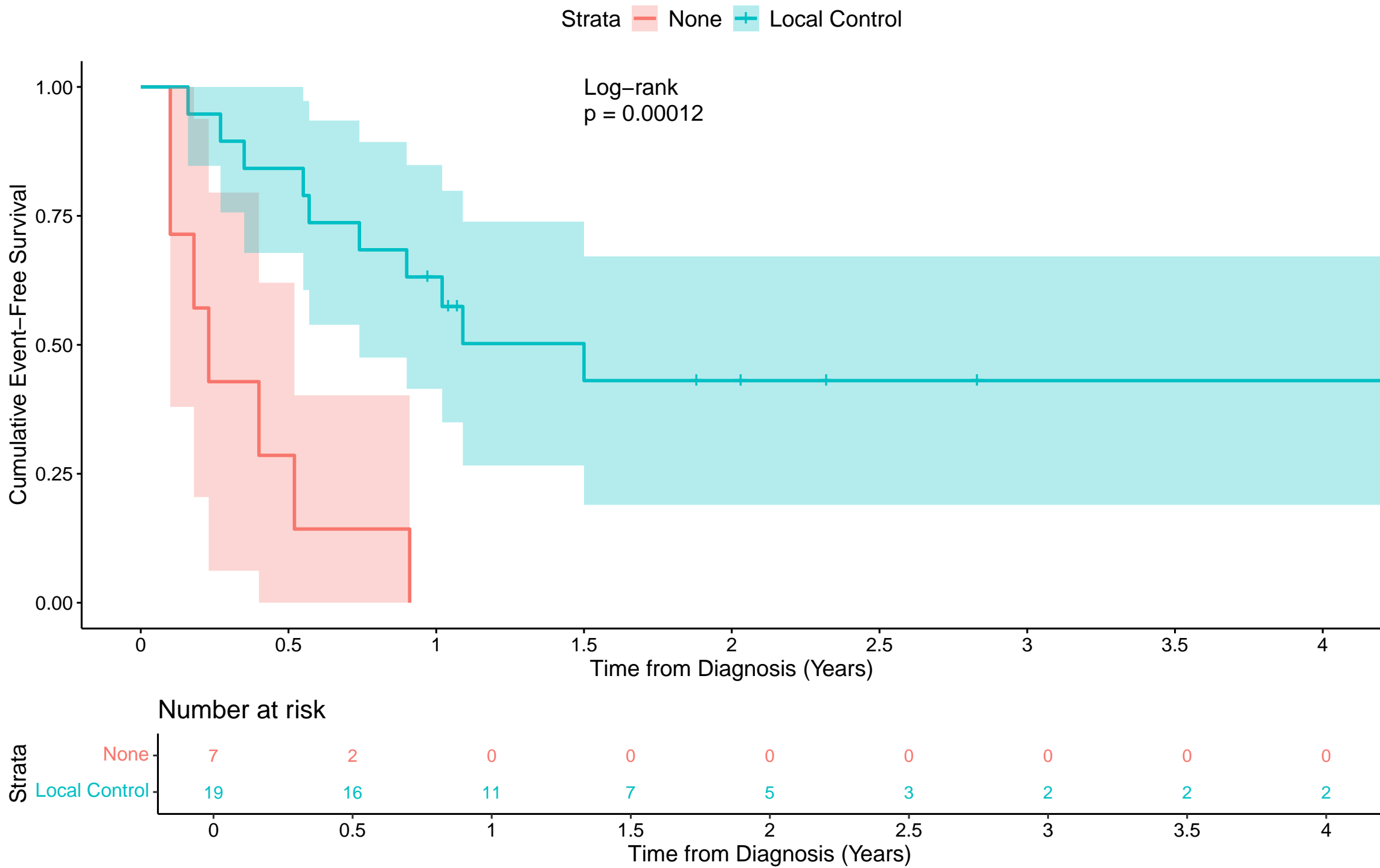

Supplement: Supplementary file 2 — Figure S2: Kaplan–Meier analysis of 2‐year event‐free survival stratified by treatment modality. [file CAM4-15-e71495-s001.zip › cam471495-sup-0003-FigureS2@suppfig2A.pdf]

**B**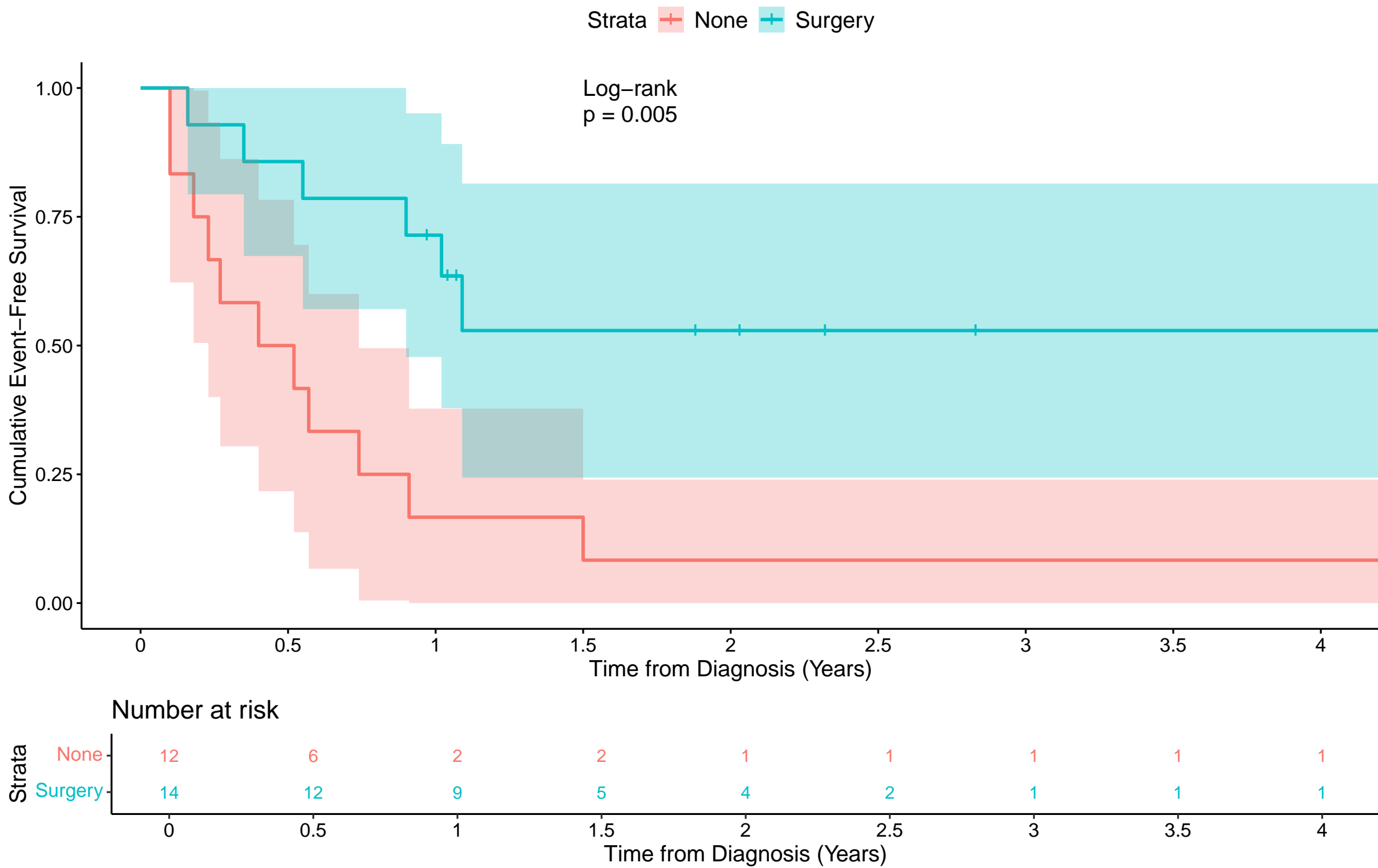

Supplement: Supplementary file 2 — Figure S2: Kaplan–Meier analysis of 2‐year event‐free survival stratified by treatment modality. [file CAM4-15-e71495-s001.zip › cam471495-sup-0004-FigureS2@suppfig2B.pdf]

**c**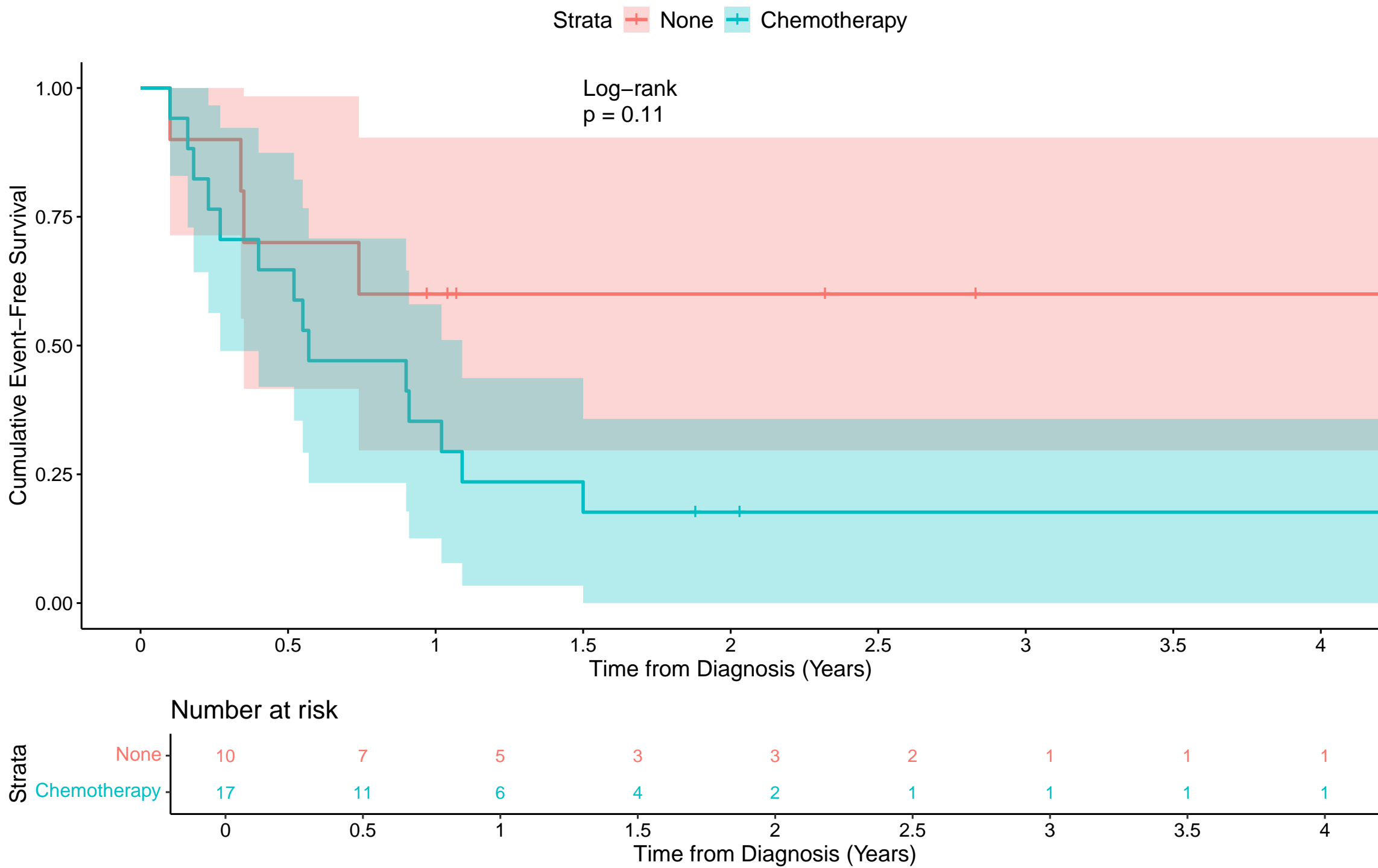

Supplement: Supplementary file 2 — Figure S2: Kaplan–Meier analysis of 2‐year event‐free survival stratified by treatment modality. [file CAM4-15-e71495-s001.zip › cam471495-sup-0005-FigureS2@suppfig2C.pdf]

**c**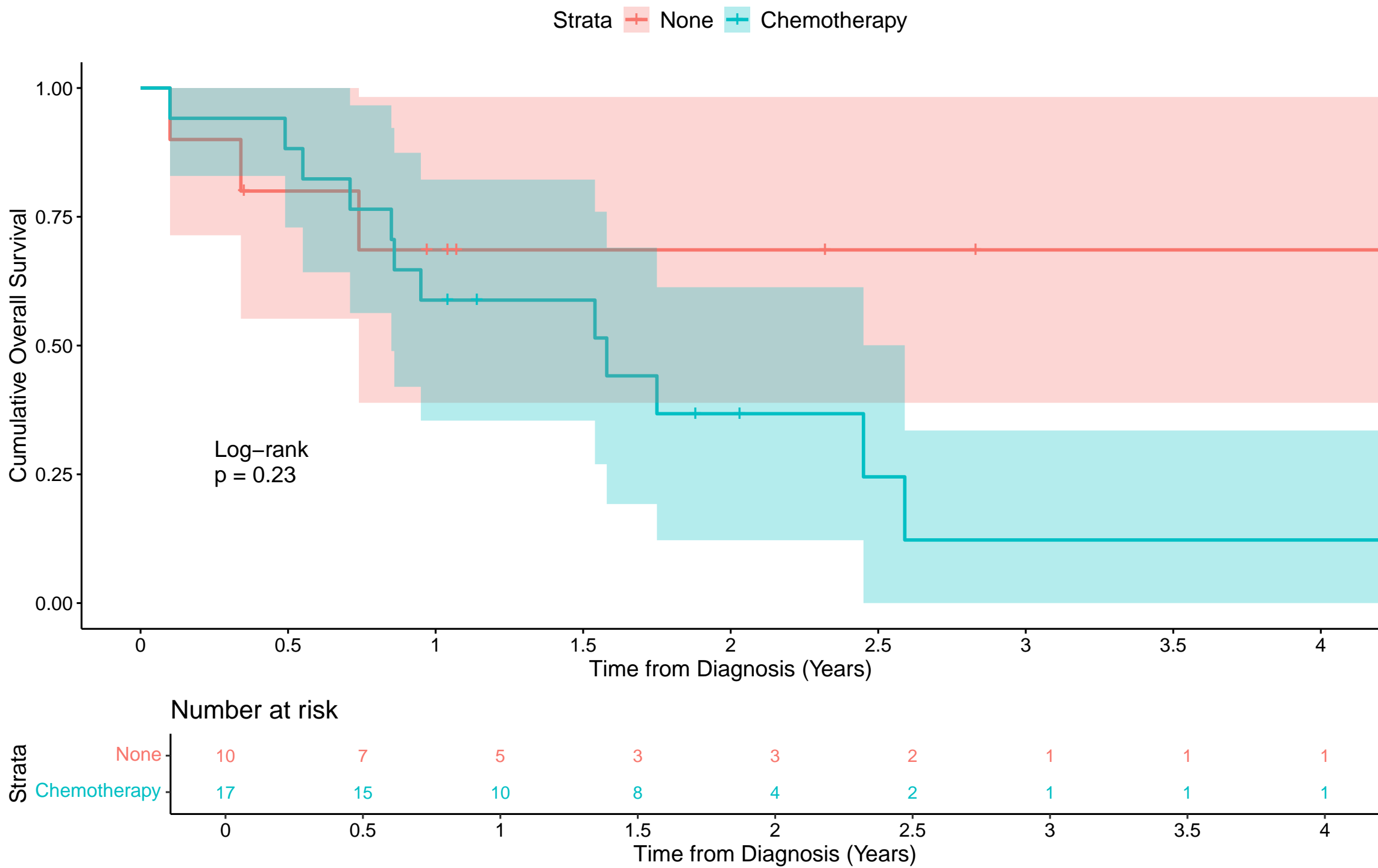

Supplement: Supplementary file 3 — Figure S3: Kaplan–Meier analysis of 2‐year overall survival stratified by treatment modality. [file CAM4-15-e71495-s004.zip › cam471495-sup-0008-FigureS3@suppfig3C.pdf]

**A**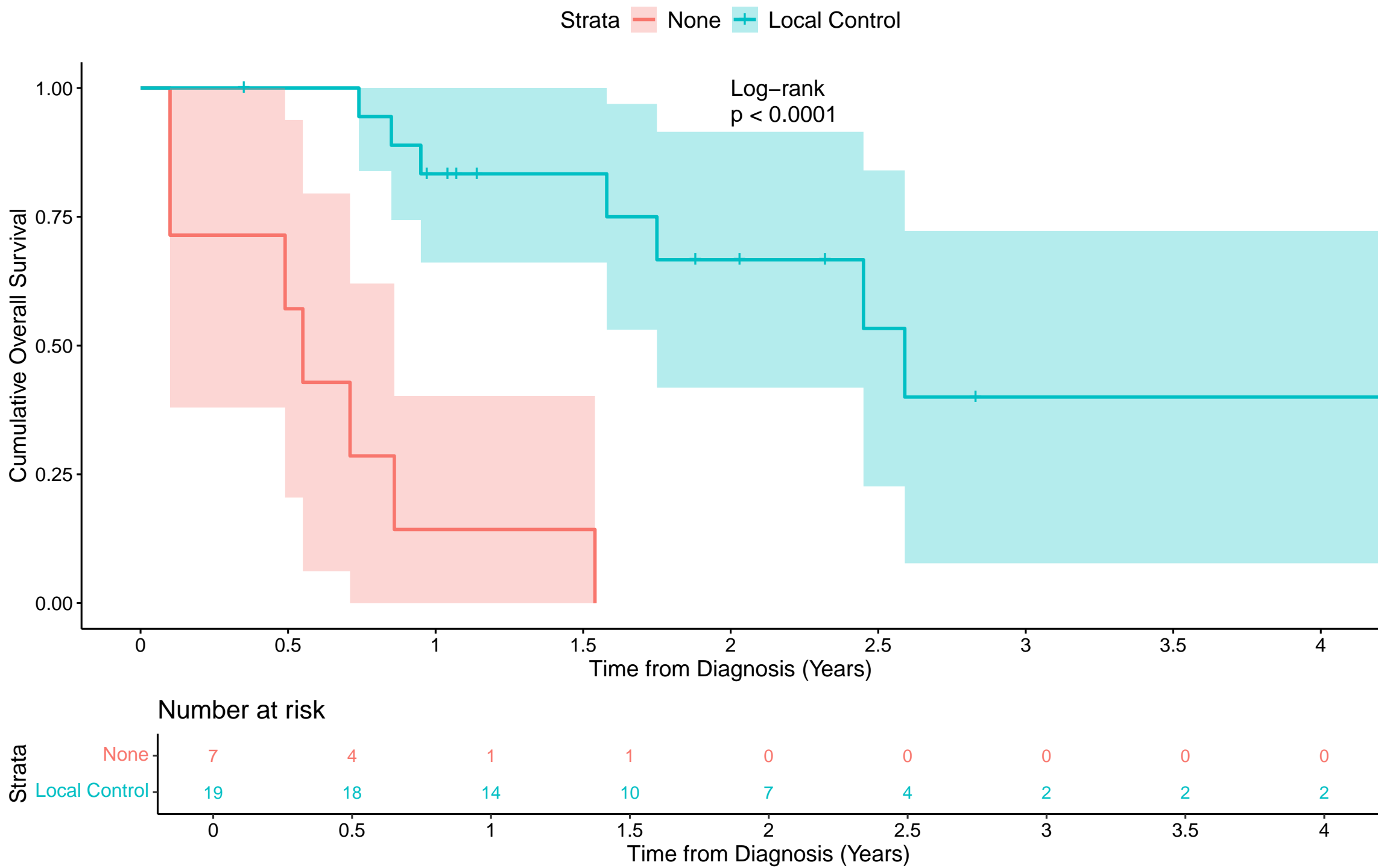

Supplement: Supplementary file 3 — Figure S3: Kaplan–Meier analysis of 2‐year overall survival stratified by treatment modality. [file CAM4-15-e71495-s004.zip › cam471495-sup-0006-FigureS3@suppfig3A.pdf]

**B**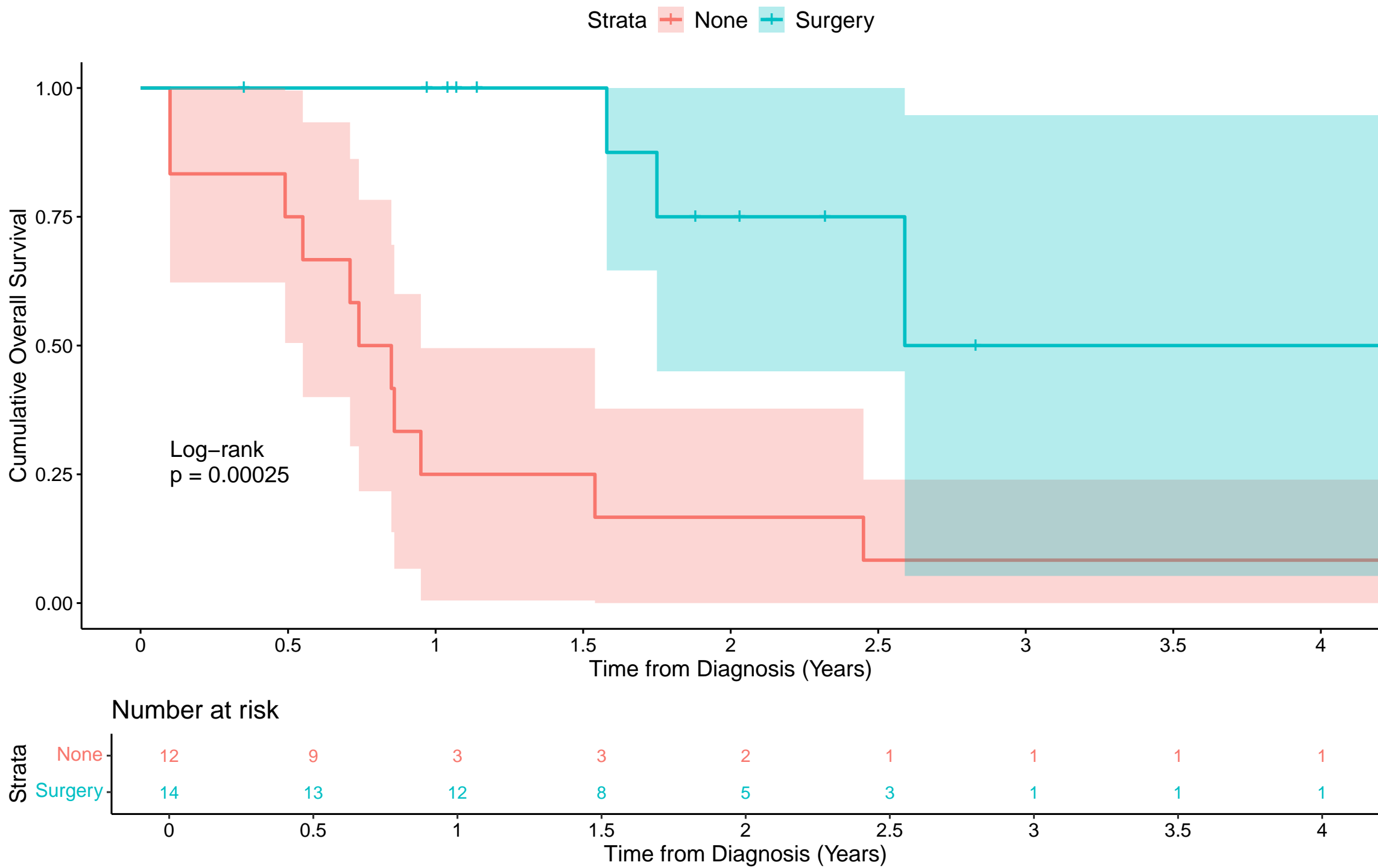

Supplement: Supplementary file 3 — Figure S3: Kaplan–Meier analysis of 2‐year overall survival stratified by treatment modality. [file CAM4-15-e71495-s004.zip › cam471495-sup-0007-FigureS3@suppfig3B.pdf]

**A**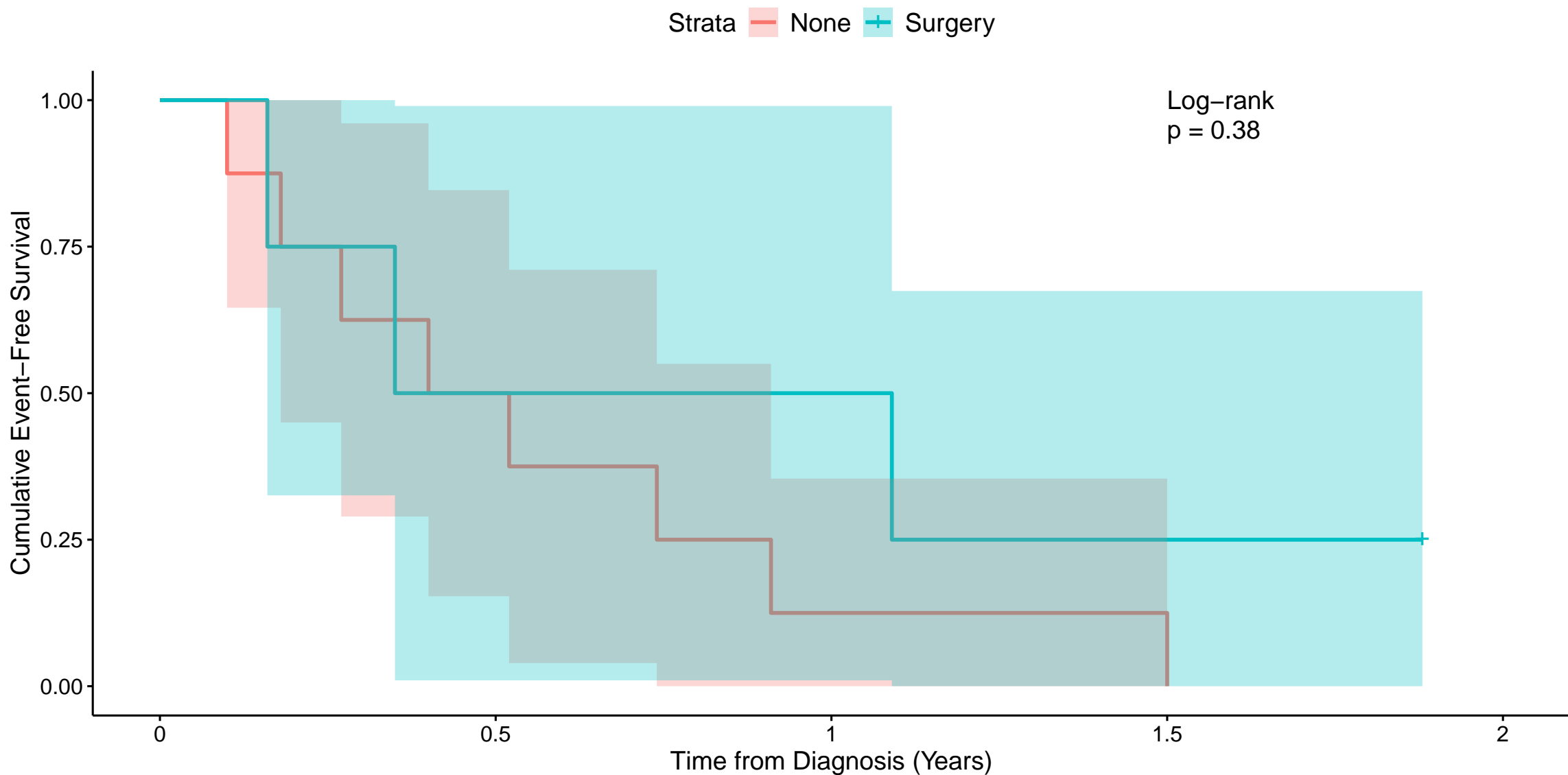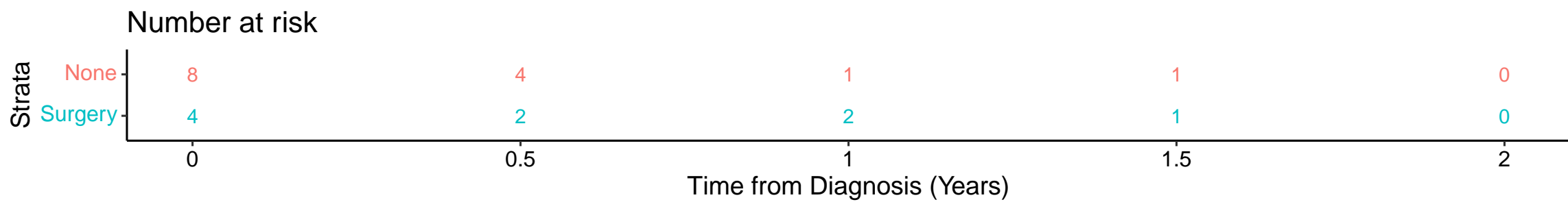

Supplement: Supplementary file 4 — Figure S4: Kaplan–Meier estimates of 2‐year event‐free survival and overall survival stratified by definitive surgery, adjusted for metastatic status. [file CAM4-15-e71495-s005.zip › cam471495-sup-0009-FigureS4@suppfig4A.pdf]

**B**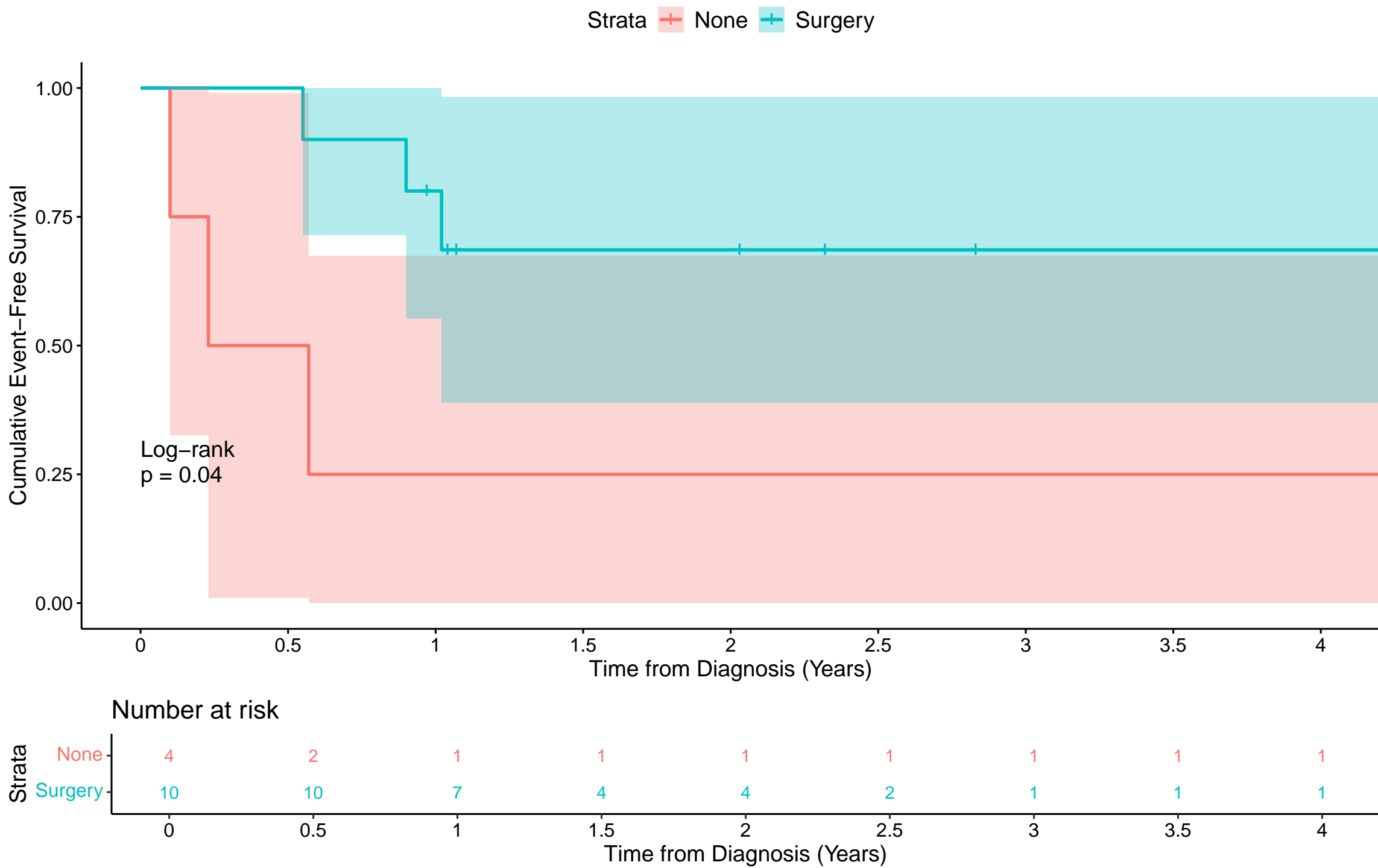

Supplement: Supplementary file 4 — Figure S4: Kaplan–Meier estimates of 2‐year event‐free survival and overall survival stratified by definitive surgery, adjusted for metastatic status. [file CAM4-15-e71495-s005.zip › cam471495-sup-0010-FigureS4@suppfig4B.pdf]

**c**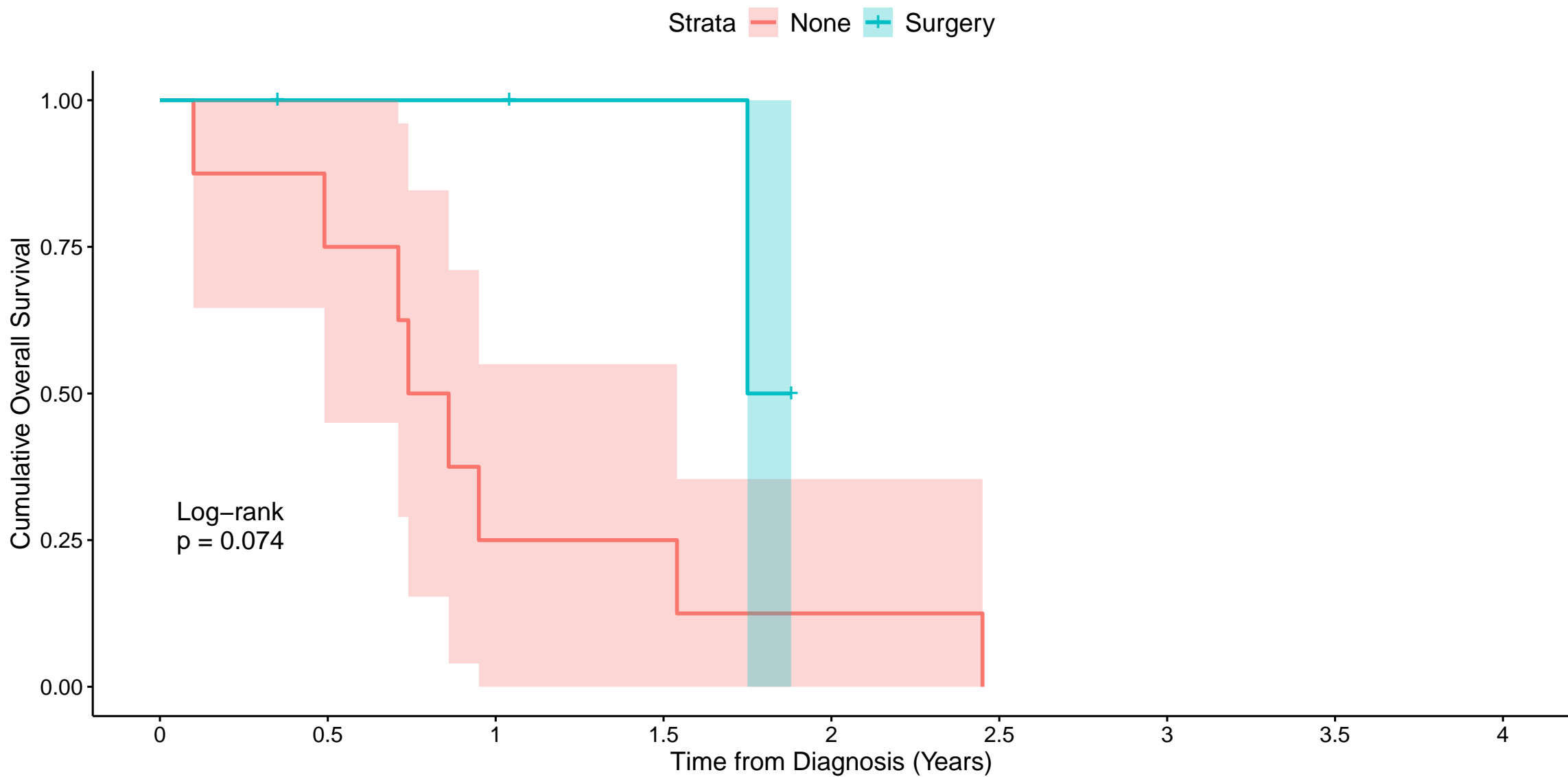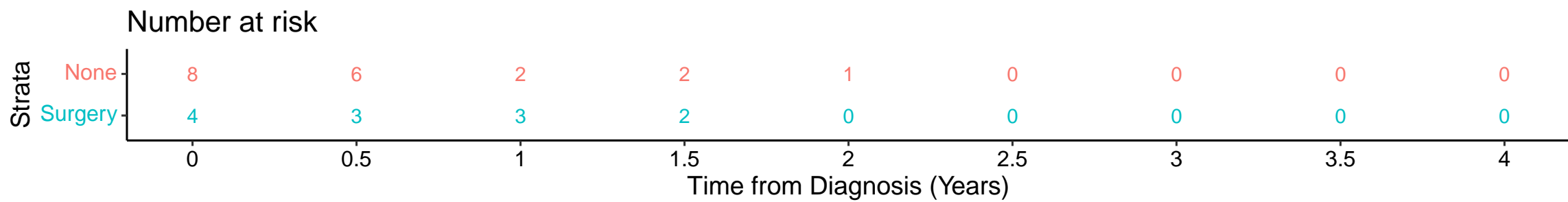

Supplement: Supplementary file 4 — Figure S4: Kaplan–Meier estimates of 2‐year event‐free survival and overall survival stratified by definitive surgery, adjusted for metastatic status. [file CAM4-15-e71495-s005.zip › cam471495-sup-0011-FigureS4@suppfig4C.pdf]

**D**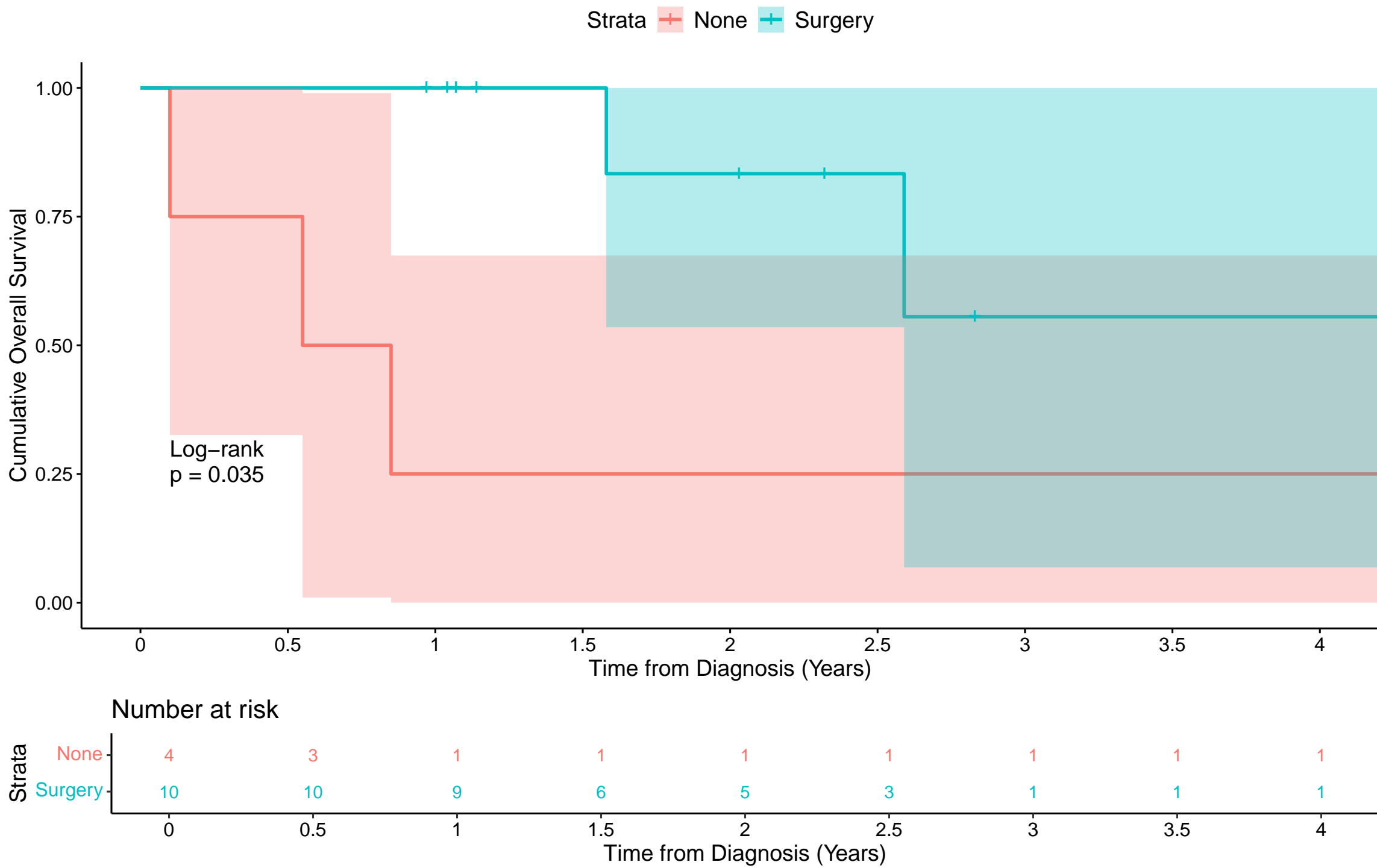

Supplement: Supplementary file 4 — Figure S4: Kaplan–Meier estimates of 2‐year event‐free survival and overall survival stratified by definitive surgery, adjusted for metastatic status. [file CAM4-15-e71495-s005.zip › cam471495-sup-0012-FigureS4@suppfig4D.pdf]

**D**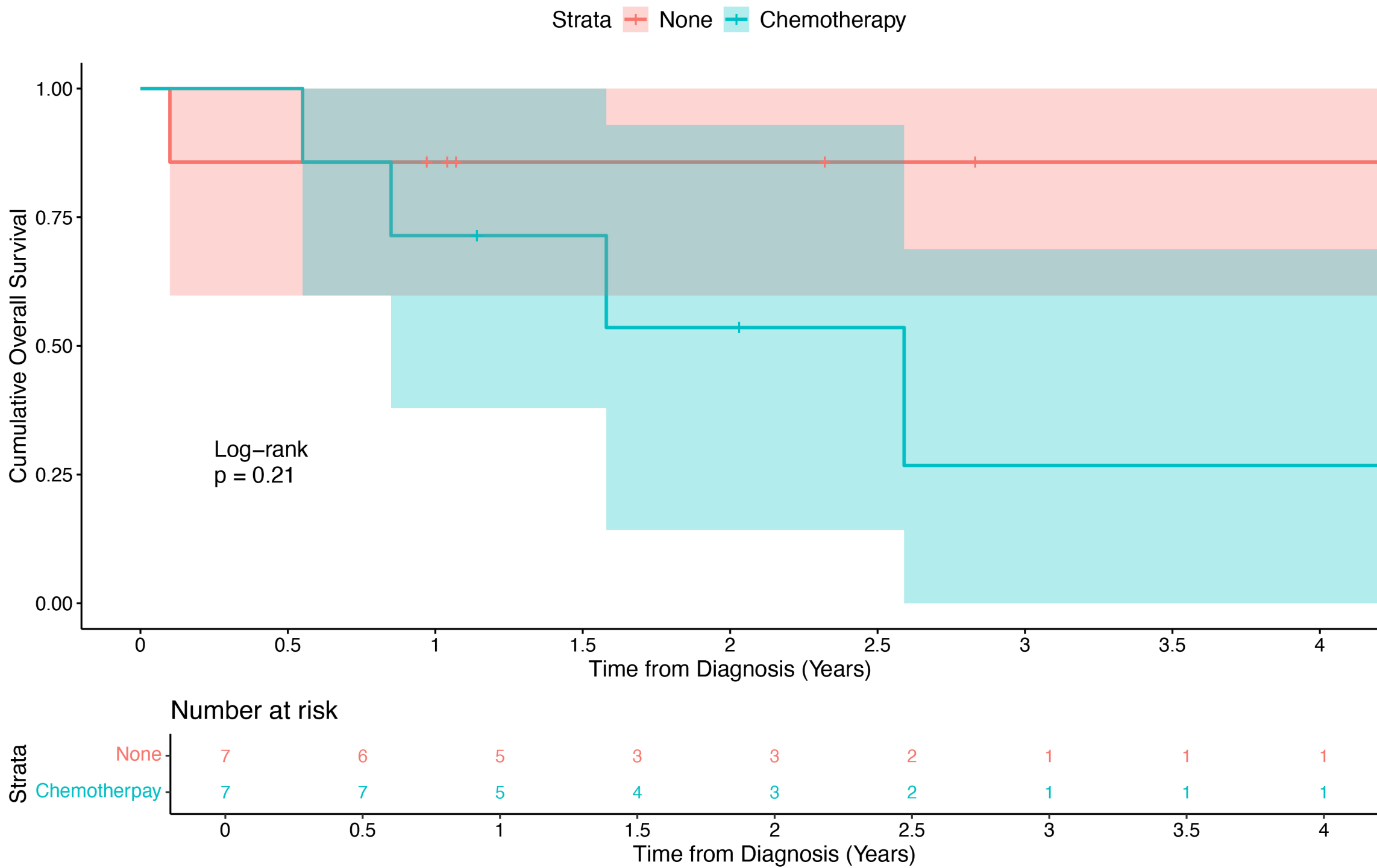

Supplement: Supplementary file 5 — Figure S5: Kaplan–Meier estimates of 2‐year event‐free survival and overall survival stratified by chemotherapy use, adjusted for metastatic status. [file CAM4-15-e71495-s002.zip › cam471495-sup-0016-FigureS5@suppfig5D.pdf]

**A**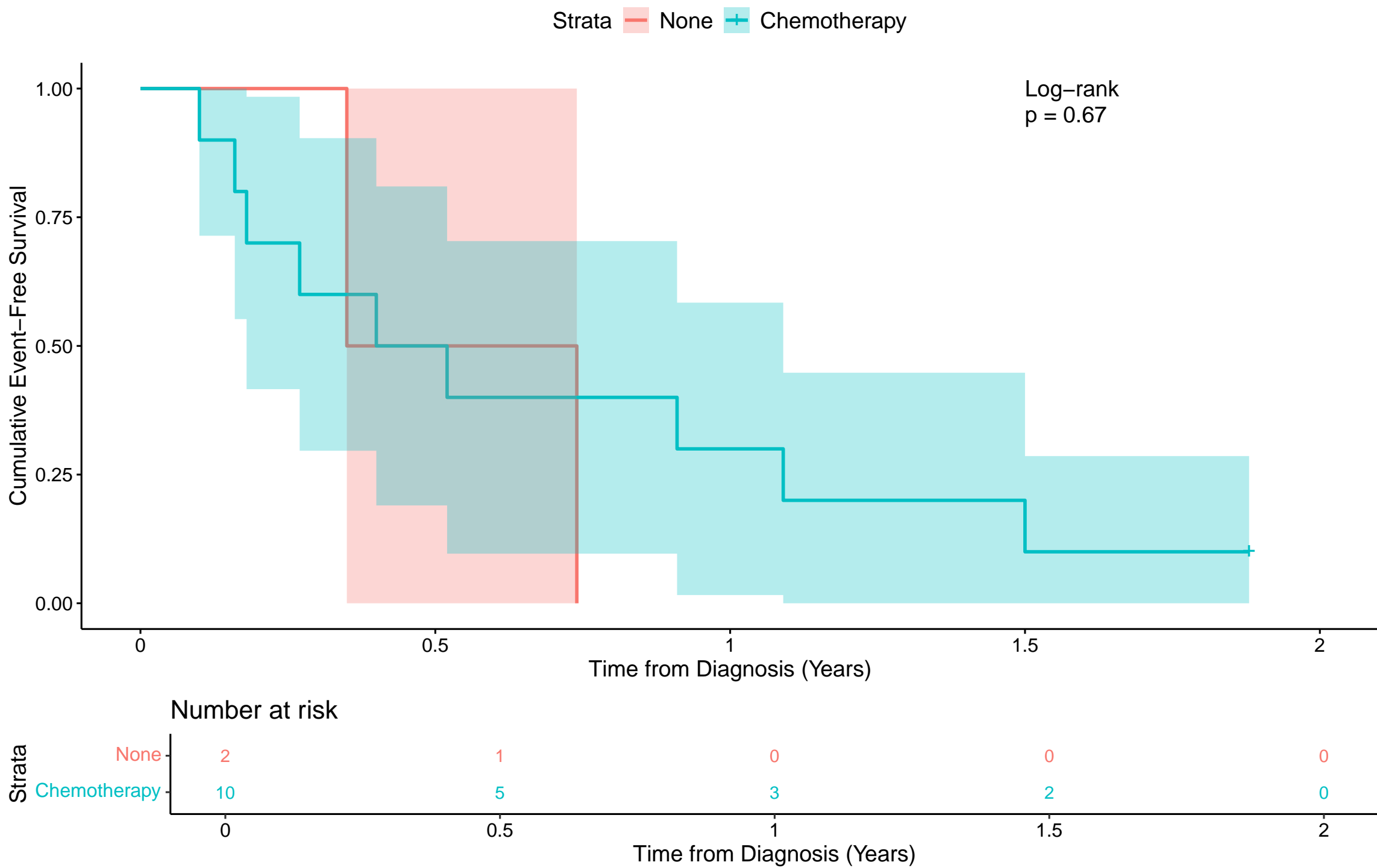

Supplement: Supplementary file 5 — Figure S5: Kaplan–Meier estimates of 2‐year event‐free survival and overall survival stratified by chemotherapy use, adjusted for metastatic status. [file CAM4-15-e71495-s002.zip › cam471495-sup-0013-FigureS5@suppfig5A.pdf]

**B**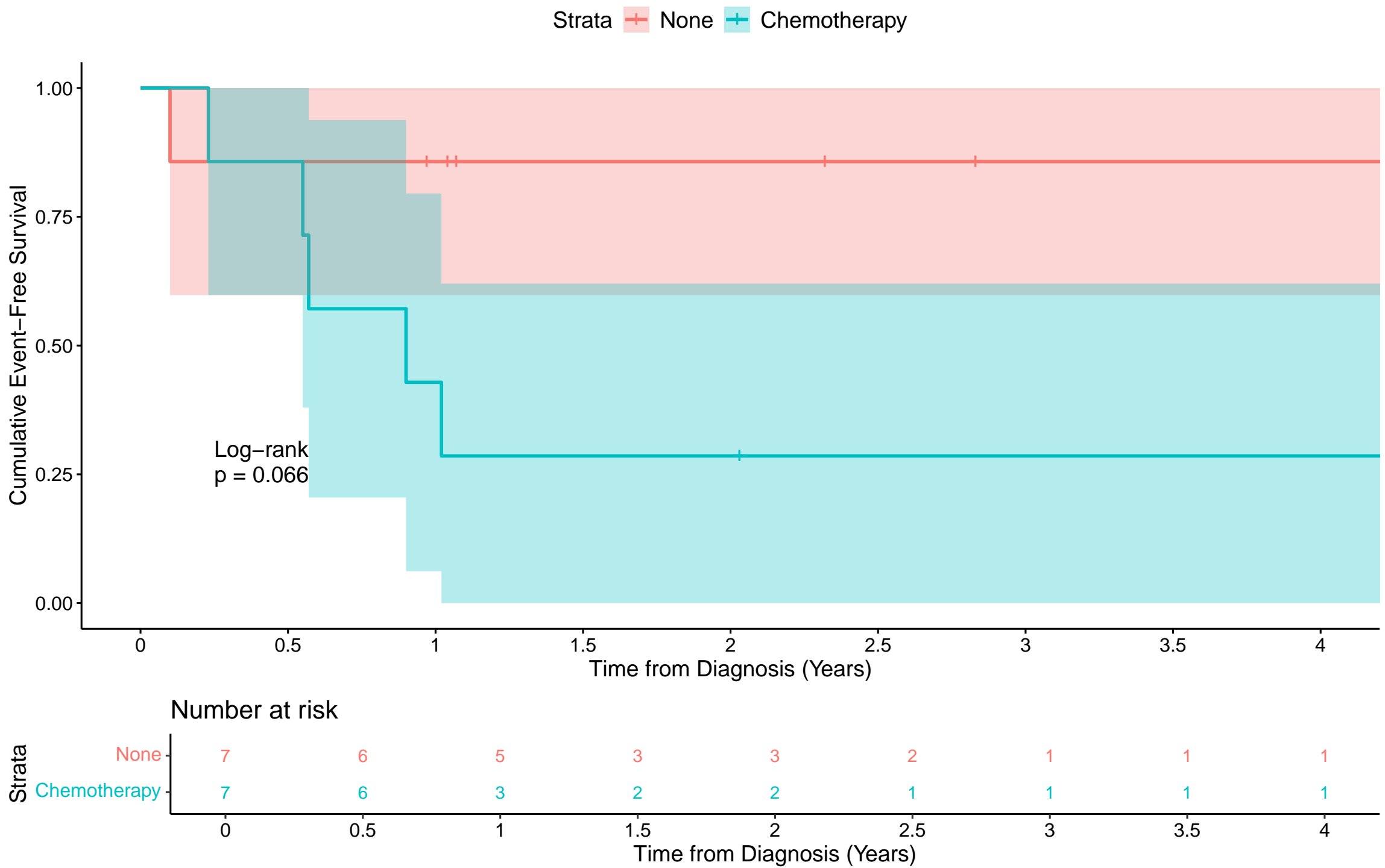

Supplement: Supplementary file 5 — Figure S5: Kaplan–Meier estimates of 2‐year event‐free survival and overall survival stratified by chemotherapy use, adjusted for metastatic status. [file CAM4-15-e71495-s002.zip › cam471495-sup-0014-FigureS5@suppfig5B.pdf]

**c**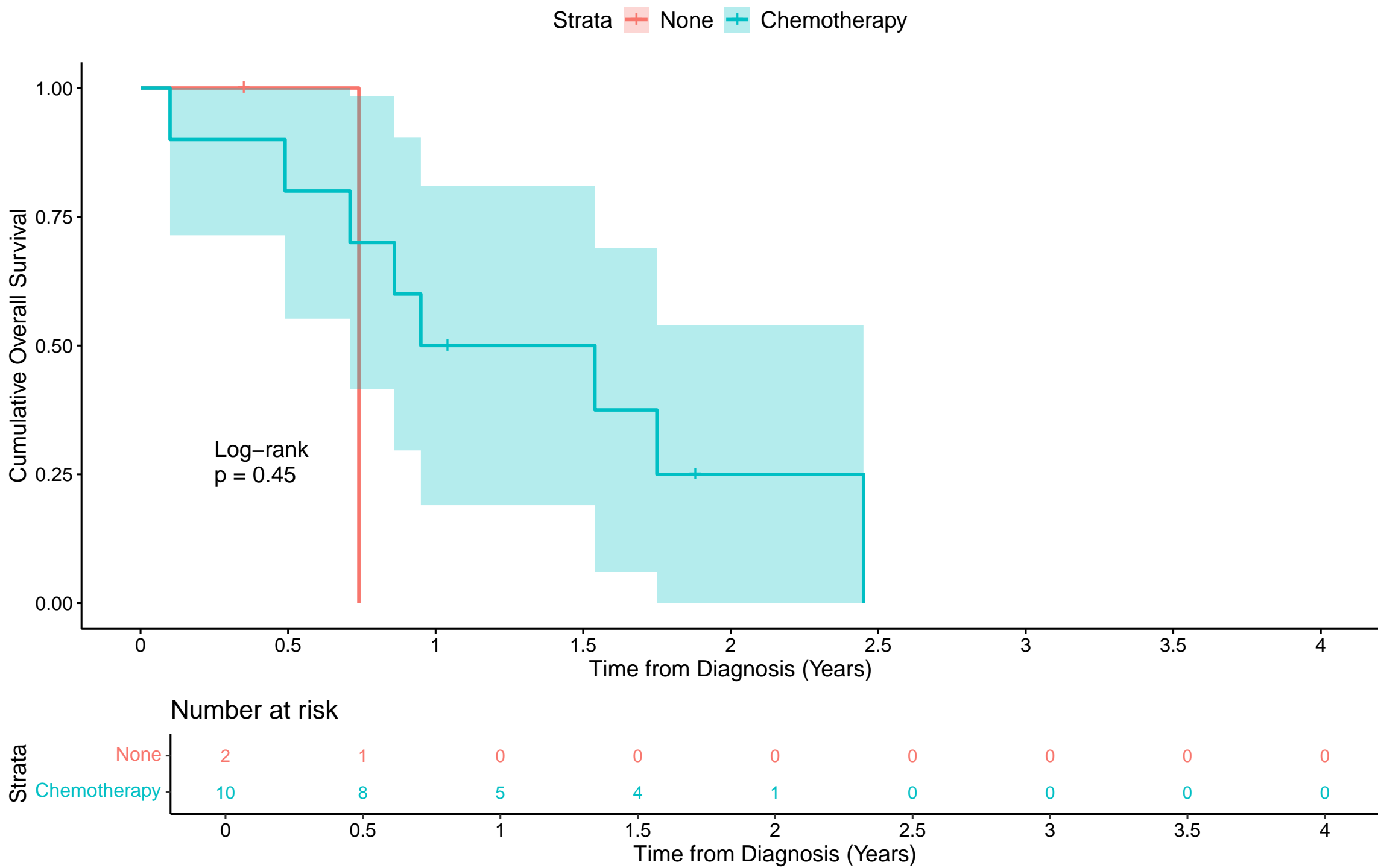

Supplement: Supplementary file 5 — Figure S5: Kaplan–Meier estimates of 2‐year event‐free survival and overall survival stratified by chemotherapy use, adjusted for metastatic status. [file CAM4-15-e71495-s002.zip › cam471495-sup-0015-FigureS5@suppfig5C.pdf]
