# Supplementary material for: A Real‐World Analysis of Outcomes in CIC‐Rearranged Sarcomas: A Canadian Sarcoma Research and Clinical Collaboration (CanSaRCC) Study
Source: Cancer Med. 2026 Jan 19;15(1):e71495. doi: 10.1002/cam4.71495 (PMC12815609; doi:10.1002/cam4.71495)
Supplement: Supplementary file 6 — Table S1: Kaplan–Meier estimates and log‐rank analysis of event‐free and overall survival in patients with CIC‐rearranged sarcoma. [file CAM4-15-e71495-s003.docx]

**SUPPLEMENTARY MATERIAL**

**Supplementary Table 1. Kaplan-Meier estimates and log-rank analysis of event-free and overall survival in patients with CIC-rearranged sarcoma.**

|  | 2-Year EFS % (95% CI) | Log-rank p-Value | 2-Year OS % (95% CI) | Log-rank p-Value |
| --- | --- | --- | --- | --- |
| Total | 30 (12-49) | - | 45 (24-66) | - |
| Metastatic disease at diagnosis (Yes vs. No) | 8 (0-24) vs. 56 (30-83) | 0.02 | 23 (0-50) vs. 67 (40-95) | 0.02 |
| Local control (No vs. Yes)   - Loc: - Met: | 0 (0-0) vs. 43 (19-67)  0 (0-0) vs. 66 (38-93)  0 (0-0) vs. 14 (0-40) | <0.001  0.01* | 0 (0-0) vs. 67 (42-92)  0 (0-0) vs. 79 (51-100)  0 (0-0) vs. 44 (1-88) | <0.01  <0.01* |
| Surgery (No vs. Yes)   - Localized: - Metastatic: | 8 (0-22) vs. 57 (27-87)  20 (0-55) vs. 76 (47-100)  0 (0-0) vs. 25 (0-68) | <0.01  0.03* | 23 (0-46) vs. 71 (38-100)  40 (0-82) vs. 80 (45-100)  13 (0-35) vs. 50 (0-100) | <0.01  <0.01* |
| Chemotherapy (No vs. Yes) | 55 (25-84) vs. 19 (0-38) | 0.18 | 72 (44-99) vs. 32 (13-57) | 0.19 |

Two-year EFS and OS rates with 95% CI are presented for the full cohort and stratified subgroups. P values from log-rank tests compare survival distributions. Where indicated (*), survival comparisons are adjusted for metastatic status at diagnosis.

**Abbreviations:** OS = overall survival; CI = confidence interval; EFS = event-free survival; vs. = versus.

**Supplementary Figure 1. Kaplan-Meier estimates of 2-year event-free and overall survival in patients with CIC-rearranged sarcoma.**

Kaplan-Meier (KM) estimate of 2-year EFS: 30% (95% CI: 12-49%). (B) KM estimate of 2-year overall survival (OS): 45% (95% CI: 24-66%). Tick marks indicate censored observations.

**Abbreviations:** KM = Kaplan-Meier; EFS = event-free survival; CI = confidence interval.

**Supplementary Figure 2**. **Kaplan-Meier analysis of 2-year event-free survival stratified by treatment modality.**

(A) Stratification by receipt of local control (surgery and/or radiotherapy): No = 0% (95% CI: 0-0%) vs. Yes = 43% (95% CI: 19-67%); *P*<0.001. (B) Stratification by definitive surgery for local control: No = 8% (95% CI: 0-22%) vs. Yes = 57% (95% CI: 27-87%); *P*<0.01. (C) Stratification by chemotherapy: No = 55% (95% CI: 25-84%) vs. Yes = 19% (95% CI: 0-38%); *P*=0.18. Tick marks indicate censored observations.

**Abbreviations:** CI – confidence interval.

**Supplementary Figure 3. Kaplan-Meier analysis of 2-year overall survival stratified by treatment modality.**

(A) Stratification by receipt of local control (surgery and/or radiotherapy): No = 0% (95% CI: 0-0%) vs. Yes = 67% (95% CI 42-92%); *P*<0.01. (B) Stratification by definitive surgery for local control: No = 23% (95% CI: 0-46%) vs. Yes = 71% (95% CI: 38-100%); *P*<0.01. (C) Stratification by chemotherapy: No = 72% (95% CI: 44-99%) vs. Yes = 32% (95% CI: 13-57%); *P*=0.19. Tick marks indicate censored observations

**Abbreviations:** CI – confidence interval.

**Supplementary Figure 4. Kaplan-Meier estimates of 2-year event-free survival and overall survival stratified by definitive surgery, adjusted for metastatic status.**

(A) EFS stratified by definitive surgery among patients with metastatic disease at diagnosis: No = 0% (95% CI: 0–0%) vs. Yes = 25% (95% CI: 0–68%); *P*=0.38. (B) EFS stratified by definitive surgery among patients with localized disease at diagnosis: No = 20% (95% CI: 0–55%) vs. Yes = 76% (95% CI: 47–100%); *P*<0.01. (C) OS stratified by definitive surgery among patients with metastatic disease at diagnosis: No = 12% (95% CI: 0–35%) vs. Yes = 50% (95% CI: 0–100%); *P*=0.07. (D) OS stratified by definitive surgery among patients with localized disease at diagnosis: No = 40% (95% CI: 0–82%) vs. Yes = 80% (95% CI: 45–100%); *P*=0.03. Tick marks indicate censored observations.

**Abbreviations:** EFS = event-free survival; OS = overall survival; CI = confidence interval.

**Supplementary Figure 5. Kaplan-Meier estimates of 2-year event-free survival and overall survival stratified by chemotherapy use, adjusted for metastatic status.**

(A) EFS among patients with metastatic disease: No chemotherapy = 0% (95% CI: 0–0%) vs. Yes = 10% (95% CI: 0–29%); *P*=0.67.

(B) EFS among patients with localized disease: No chemotherapy = 75% (95% CI: 45–100%) vs. Yes = 33% (95% CI: 0–71%); *P*=0.23.

(C) OS among patients with metastatic disease: No chemotherapy = 0% (95% CI: 0–0%) vs. Yes = 25% (95% CI: 0–54%); *P*=0.45.

(D) OS among patients with localized disease: No chemotherapy = 88% (95% CI: 65–100%) vs. Yes = 44% (95% CI: 1–88%); *P*=0.35.

Tick marks on survival curves indicate censored observations.

**Abbreviations:** EFS = event-free survival; CI = confidence interval; OS = overall survival.
